# CD73 promotes tumor metastasis by modulating RICS/RhoA signaling and EMT in gastric cancer

**DOI:** 10.1038/s41419-020-2403-6

**Published:** 2020-03-23

**Authors:** Zhijun Xu, Chuncai Gu, Xingxing Yao, Weihong Guo, Huanan Wang, Tian Lin, Fengping Li, Da Chen, Jianhua Wu, Gengtai Ye, Liying Zhao, Yanfeng Hu, Jiang Yu, Jiaolong Shi, Guoxin Li, Hao Liu

**Affiliations:** 1 Department of General Surgery, Nanfang Hospital, Southern Medical University, Guangzhou, China; 2 Department of Gastroenterology, Nanfang Hospital, Southern Medical University, Guangzhou, China; 3 Department of Oncology, Nanfang Hospital, Southern Medical University, Guangzhou, China

**Keywords:** Cancer microenvironment, Metastasis

## Abstract

Tumor microenvironment plays vital roles in shaping cancer diversity, and CD73 (ecto-5′-nucleotidase; NT5E) is an emerging immune checkpoint in modulating cancer progression via conversion of immunostimulatory ATP into immunosuppressive adenosine. However, how the CD73 is regulated and how it functions in the progression of cancer are largely unknown. Here, we showed that CD73 was overexpressed and correlated with poor prognosis of gastric cancer. CD73 links adenosinergic signaling in microenvironment switching to induction of epithelial-to-mesenchymal transition phenotype in gastric cancer during metastasis. Further pathway and gene set enrichment analysis of transcriptome data revealed the modulation role of CD73 in RICS/RhoA signaling by its extracellular function in adenosinergic pathway, which subsequently inhibited phosphorylation of LIMK/cofilin and promoted β-catenin activation. Pharmacological inhibition of CD73 adenosinergic signaling was found to induce RICS dysfunction. Dissemination and hematogenous metastasis model showed that targeting CD73 in gastric cancer could suppress experimental metastasis. To conclude, it substantiates CD73 as a target for treatment of gastric cancer metastasis and verifies RICS as an intracellular functional molecule linking CD73/adenosinergic signaling switching to RhoA/LIMK/cofilin pathway.

## Introduction

Gastric cancer (GC) represents a major health burden worldwide^1–3^. Metastatic GC is a lethal disease characterized by a very poor survival, underlining a critical need for metastasis mechanics research^4^. Functional subsets of cancer cells from the heterogeneous tumor with specific gene signature and invasive phenotype should be defined to understand deeply about the dissemination process of GC and provide more effective targets for treatment^5–7^.

Extensive research has reported that purinergic signaling is a crucial component of tumor microenvironments (TMEs) with therapeutic potential in solid tumors^8,9^. Purine or pyrimidine nucleotides, such as ATP, ADP, and adenosine in TMA, have been discovered as important signaling molecules that engaged different P1 nucleoside and P2 nucleotide receptors on cancer or stromal cells^10–13^. The complex network of purinergic signaling events plays a key role in immune escape and accelerated growth of cancer cells, and eventually metastasis. Therefore, abolishing the production of purine nucleotides or blocking the intimate associations between nucleotides and purinoceptors may be a promising therapy for cancer.

CD73 (ecto-5′-nucleotidase; NT5E), a membrane-bound and soluble homodimer that catalyzes the conversion of extracellular AMP to membrane-permeable nucleosides, is a critical responder of oxygen deprivation or inflammation and also powerful immunosuppressor in maintaining tumor survival^14^. Growing evidence has demonstrated the vital role of CD73 in TME, as a rate-limiting enzyme in the production of extracellular adenosine and modulator of immune cells and responses^15^. Both tumor and host CD73 overexpression have been observed in multiple types of human cancer. Distinct roles of adenosinergic effects in regulating systemic or local antitumor T cell responses were well illustrated. CD73 on nonhematopoietic cells limited antitumor T cells homing to tumors in multiple mouse model, while CD73 on hematopoietic cells induced dysfunction of systemic antitumor T cell expansion and effector^16^. Additionally, CD73 was verified to be involved in carcinogenesis, cancer apoptosis escape, and therapeutic resistance^15,17–19^. From a clinical meta-analysis, CD73 expression level was identified in distinct cancers and verified as independent prognostic indicator for gastric carcinoma^20,21^. However, whether CD73 is involved in the progression of gastric cancer and the underlying mechanism remains unclear.

In this study, we report that CD73 is overexpressed in GC patients, especially in those with more progressive pathological features or metastatic properties. Overexpression of CD73 is an independent indicator of poor prognosis in GC patients. In vitro induction or suppression of CD73 reveals an essential role of CD73 in GC cell migration and invasion. Further mechanism study suggests that CD73 links adenosinergic signaling switching to activation of RICS (ARHGAP32), a GTPase-activating protein that directly interacts with RhoA and β-catenin, thereby inhibits phosphorylation of LIMK/cofilin and promotes epithelial-to-mesenchymal transition (EMT) process. Importantly, targeting CD73 in cancer cells suppresses experimental metastasis in mouse model.

## Results

### Overexpression of CD73 is correlated with poor prognosis in GC

**Fig. 1:**
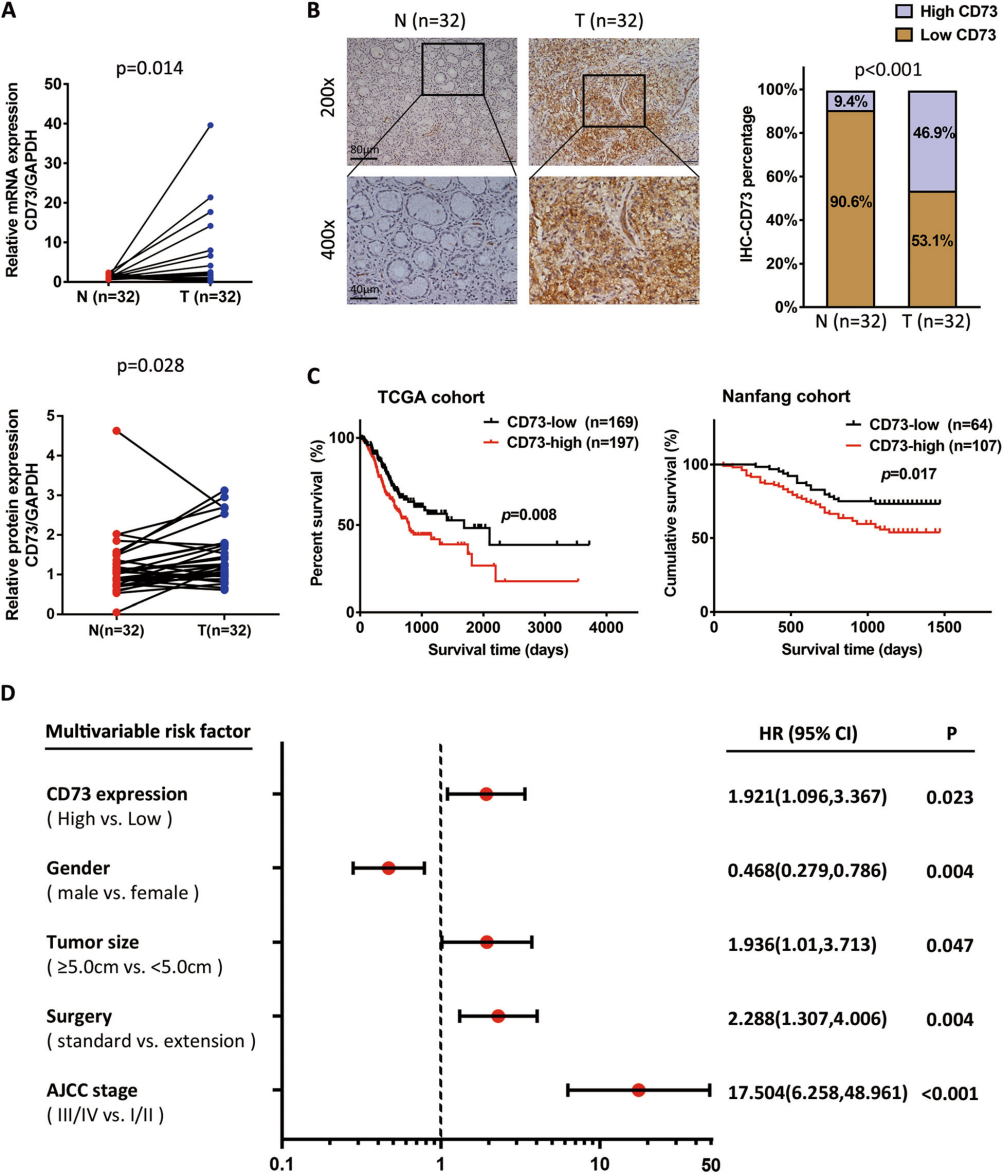
CD73 is overexpressed and correlated with poor prognosis in GC. **a** Expression of CD73 was determined by qPCR (*n* = 32) and WB (*n* = 32) in paired GC tissues separately, which was normalized by GAPDH control and analyzed with paired *t* test. **b** Protein detection of CD73 by IHC in paired GC tissues and *χ*^2^ analysis was used to compare the positive rate of CD73 expression between groups (*n* = 32). **c** Kaplan–Meier survival analysis (log-rank test) of CD73 mRNA expression in the TCGA cohort (*n* = 366) and CD73 protein expression in the Nanfang cohort (*n* = 171). **d** Multivariate analysis of significant prognostic parameters for GC patients in the Nanfang cohort.

Expression analysis of CD73 was performed in 32 pairs of human GC tissues and normal gastric mucosal tissues and it showed a significantly elevated expression of CD73 in GC patients (Fig. 1a). With subsequent morphological observation by immunohistochemical (IHC) staining, we found an obvious abundance of CD73 protein in GC tissues (Fig. 1b). To further investigate prognosis value of CD73 expression in GC patients, IHC staining was performed on a tissue microarray (TMA) containing 171 GC patients (named as Nanfang cohort) and it was found that high expression of CD73 was correlated with poor survival of GC patients (Fig. 1c). Kaplan–Meier analysis in the TCGA (The Cancer Genome Atlas) data also indicated that GC patients with high CD73 messenger RNA (mRNA) levels suffered from poor survival (Fig. 1c). With univariate and multivariate Cox proportional hazards modeling, we determined CD73 expression as an independent predictor of survival in GC patients combined with TNM stage, extension of surgery, tumor size, and gender (Fig. 1d, Supplementary Table 1). Immunofluorescence (IF) staining highlighted the overexpressed CD73 protein in the border line of cancer and adjacent normal tissues, indicating more invasive and metastasis properties of cancer (Supplementary Fig. 1A).

### CD73 promotes cancer cell migration

**Fig. 2:**
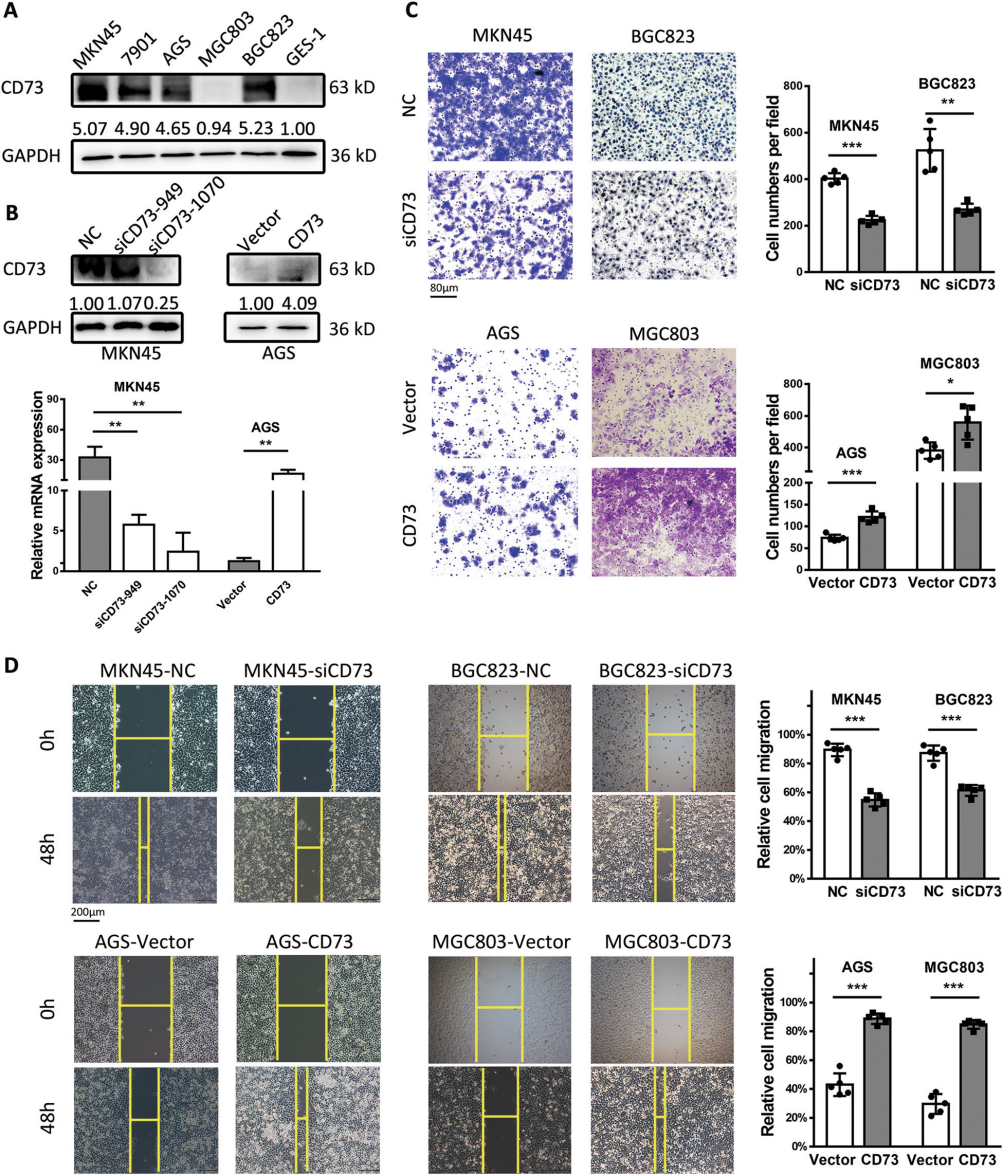
CD73 is overexpressed in GC cells and promotes GC migration in vitro. **a** Western blot analysis and relative gray value of CD73 expression in gastric cancer cell lines and GES-1. **b** Protein and mRNA levels (mean ± SD, *n* = 3, unpaired *t* test) of CD73 in MKN45 and AGS cells transfected with siRNA or overexpressed plasmids. **c** Transwell analysis of cell migration in MKN45, BGC823, AGS, and MGC803 cells transfected with siCD73 or CD73 plasmids for 24 h. The number of invaded cells in GC cells was counted and shown as mean ± SD (*n* = 5), and unpaired *t* test was used. **d** Wound healing analysis was performed to assess the migration capacity of CD73 in cancer cells and relative migration rate was shown (mean ± SD, *n* = 5, unpaired *t* test). *p < 0.05, **p < 0.01, and ***p < 0.001.

To determine the function of CD73 in GC cells, we first detected the expression level of CD73 in five human GC cell lines and a normal gastric epithelial cell GES-1, and results showed an obvious overexpression of CD73 in GC cell lines when compared with GES-1 (Fig. 2a). We transfected MKN45 cell line, which had the highest expression of CD73, with two CD73 small interfering RNAs (siRNAs) and found that siCD73-1070 was the most effective interference sequence. AGS cell line was transfected with CD73 plasmids and overexpression of CD73 was confirmed with quantitative real-time PCR (qPCR) and western blot (WB) (Fig. 2b). To detect the effect of CD73 in cancer cell migration, the transwell assay was used, and we found that CD73-knockdown GC cells showed significantly decreased migrating cells than the control group. In contrast, GC cells with CD73 overexpression exhibited more aggressive potential in migration (Fig. 2c). Moreover, wound healing assay has confirmed the findings of transwell that depletion of CD73 could significantly suppress the migration of MKN45 and BGC823, while overexpression of CD73 could enhance the invasive property of AGS and MGC803 (Fig. 2d).

**Fig. 3:**
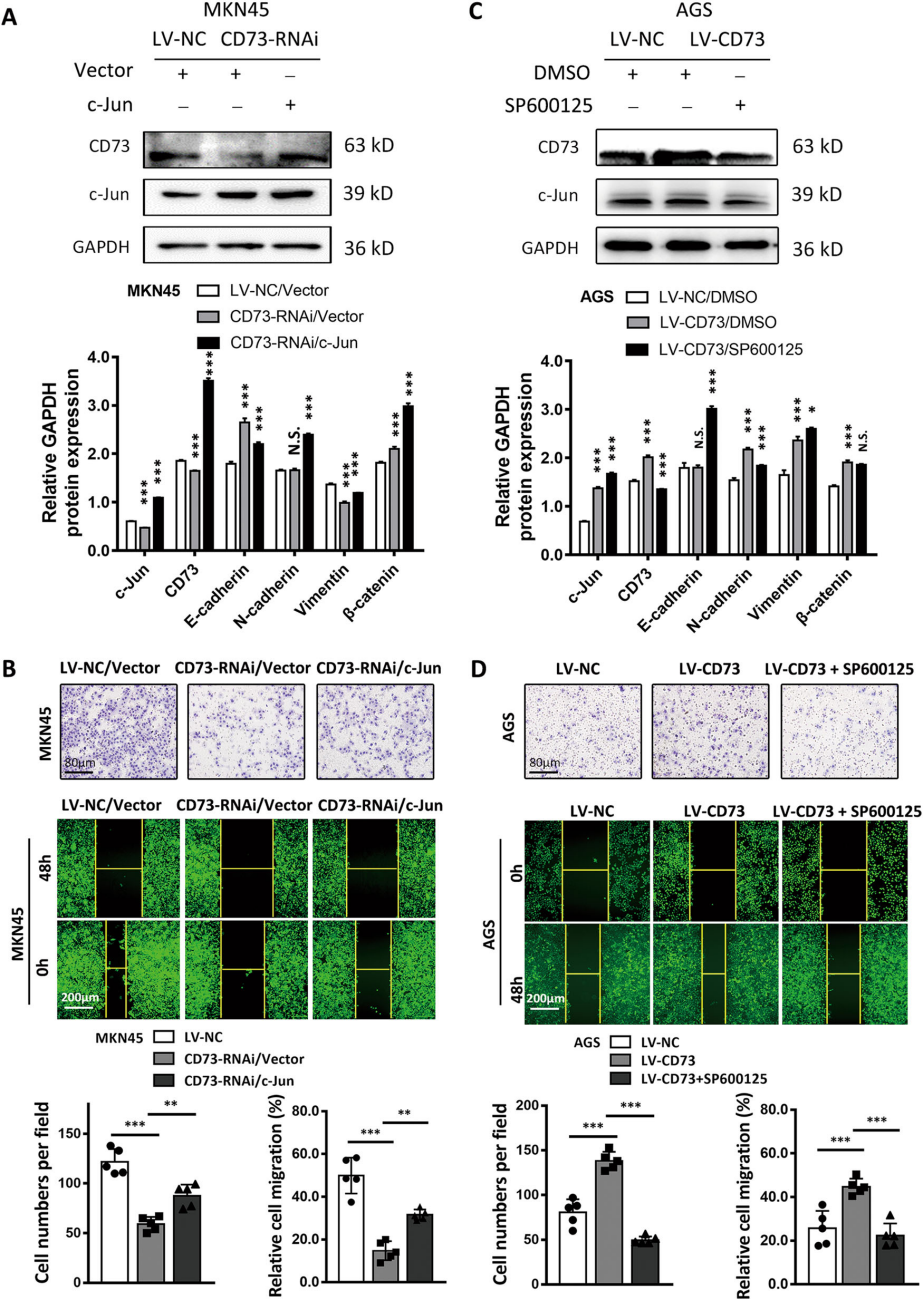
CD73-induced GC migration was regulated by c-Jun. **a**, **c** Western blot analysis of c-Jun-, CD73-, and EMT-associated proteins (E-cadherin, N-cadherin, vimentin, and β-catenin) after transfection of control vector or c-Jun for 48 h in MKN45-LV-NC or MKN45-CD73-RNAi cells and after treatment of SP600125 for 48 h in AGS-LV-CD73 cells. Relative gray value of indicated protein was shown (mean ± SD, *n* = 3, one-way ANOVA). **b**, **d** Transwell and wound healing assays were used to detect 3D and 2D migration capacity of MKN45 or AGS cells after treatment as indicated (mean ± SD, *n* = 5, one-way ANOVA). *p < 0.05, **p < 0.01, ***p < 0.001, and N.S., non-significant.

Previous studies have confirmed the intimate associations between c-Jun activation and enhanced transcription of CD73 in cancer cells; we thus investigated whether exogenous c-Jun or blockage of c-Jun phosphorylation could regulate the expression and biological function of CD73 in GC^15^. As a result, a supplement of c-Jun plasmids in MKN45-CD73-RNAi cells rescued the expression of CD73 and promoted cancer cell migration and invasion in transwell and wound healing assay (Fig. 3a, b). We corroborated this finding by using SP600125 as an inhibitor of c-Jun activation in AGS-LV-CD73 cells and verified the inhibition of CD73 as expected (Fig. 3c). Additionally, it revealed that SP600125 could abolish the invasive properties of AGS-LV-CD73 cell (Fig. 3d). Taken together, these data indicated that CD73 activated by c-Jun could induce GC migration.

### CD73 enhanced GC migration by cytoskeletal regulation and EMT process

**Fig. 4:**
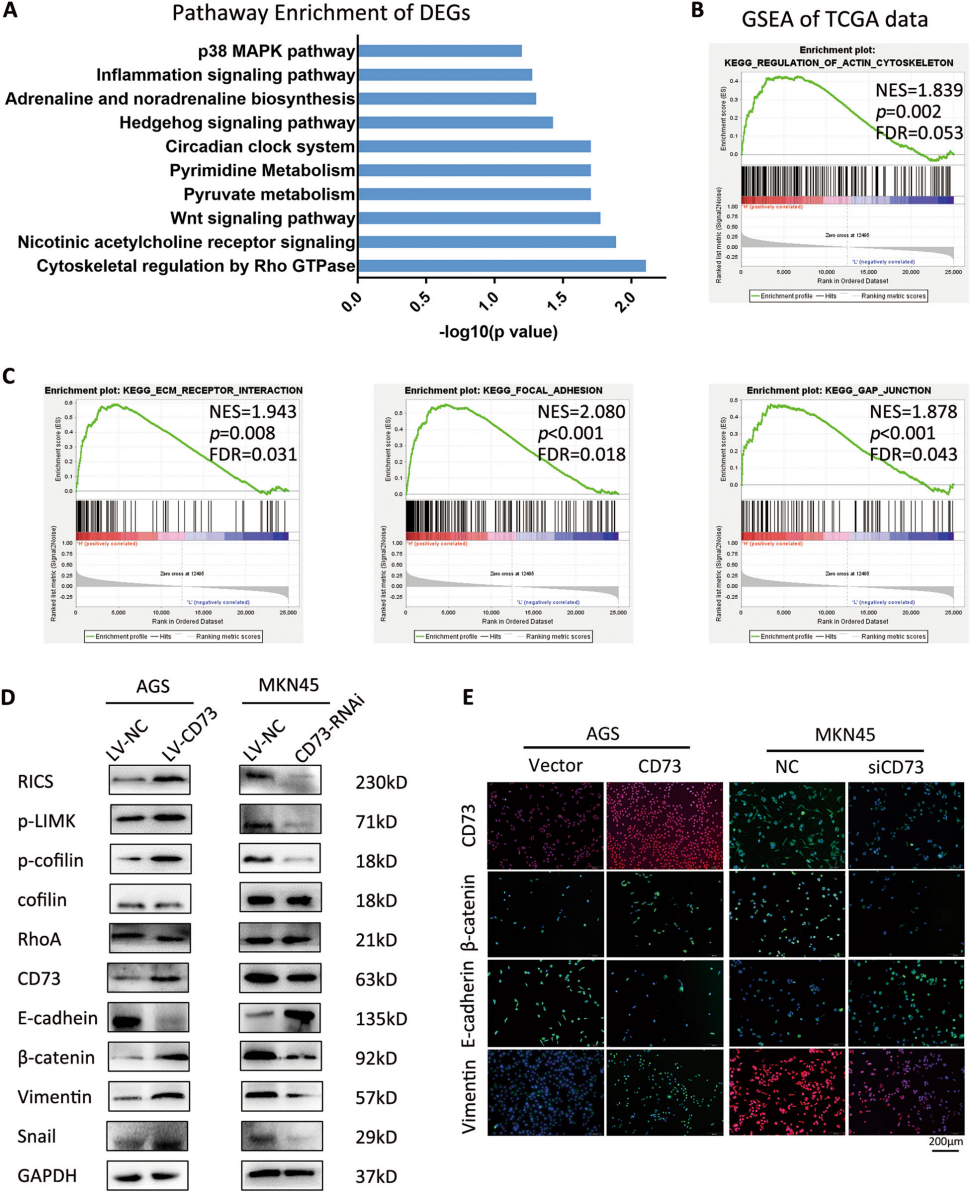
CD73 modulates cytoskeletal regulation pathway and EMT process in GC. **a** Pathway enrichment of differentially expressed genes with more than 1.5 fold change in AGS-LV-CD73 cell compared with AGS-LV-NC cell. **b** GSEA plots of top-ranking gene sets in TCGA according to the expression of CD73 and remarkable presentation of regulation of actin cytoskeleton pathway. NES, normalized enrichment score; FDR, false discovery rate. **c** GSEA plots of other cytoskeleton-associated pathways in top-ranking gene sets. **d** Western blot analysis of cytoskeleton-associated genes and EMT-associated proteins in AGS-LV-NC, AGS-LV-CD73, MKN45-LV-NC, and MKN45-CD73-RNAi cells. **e** IF staining of CD73 and EMT-associated proteins in GC cells as indicated.

To further investigate how CD73 promotes GC migration, RNA sequence was used to gain comprehensive insight into the underlying mechanism. Pathway enrichment revealed that cytoskeletal regulation by Rho GTPase pathway was enriched in AGS-LV-CD73 cell (Fig. 4a). As an external validation, we performed the gene set enrichment analysis (GSEA) of RNA sequence data from TCGA and verified that in the CD73 high group, regulation of actin cytoskeleton gene set was significantly enriched (Fig. 4b). Additionally, other gene sets related to dynamics of cytoskeleton, including ECM–receptor interaction, focal adhesion and gap junction, were significantly enriched (Fig. 4c). According to transcription results of significant genes in CD73-overexpressed cell, a crucial GTPase-activating protein, RICS, was verified to upregulate in AGS-LV-CD73 cells than in control cells (Fig. 4d). Spearman’s analysis of published GC gene expression datasets (TCGA mRNA data and public GEO data from Asian Gastric Cancers cohort, GSE36968) also showed a positive correlation between CD73 and RICS in gastric cancer samples (Supplementary Fig. 1B). Previous works presented the involvement of RICS in *N*-methyl-d-aspartate receptor signaling through its direct inhibition in RhoA activity and promotion of association between β-catenin and cadherin^22,23^. We hypothesized that CD73-induced RICS overexpression might regulate actin cytoskeleton via RhoA inactivation and β-catenin-induced EMT process. We therefore examined the RhoA/LIMK/cofilin signaling as a candidate critical pathway and EMT markers in GC cells. WB assay and qPCR detection showed elevated expression of β-catenin, mesenchyme markers including vimentin and Snail, and decreased E-cadherin in CD73-overexpressed GC cells (Fig. 4d, Supplementary Fig. 1C). An inverse alteration of EMT proteins in CD73-silenced GC cells was observed. Further IF staining in GC cells showed promoting effects of exogenous CD73 in EMT process, with consistent results observed after interference of CD73 in MKN45 (Fig. 4e).

### RICS is essential for cytoskeletal regulation and invasive properties by CD73 in GC cells

**Fig. 5:**
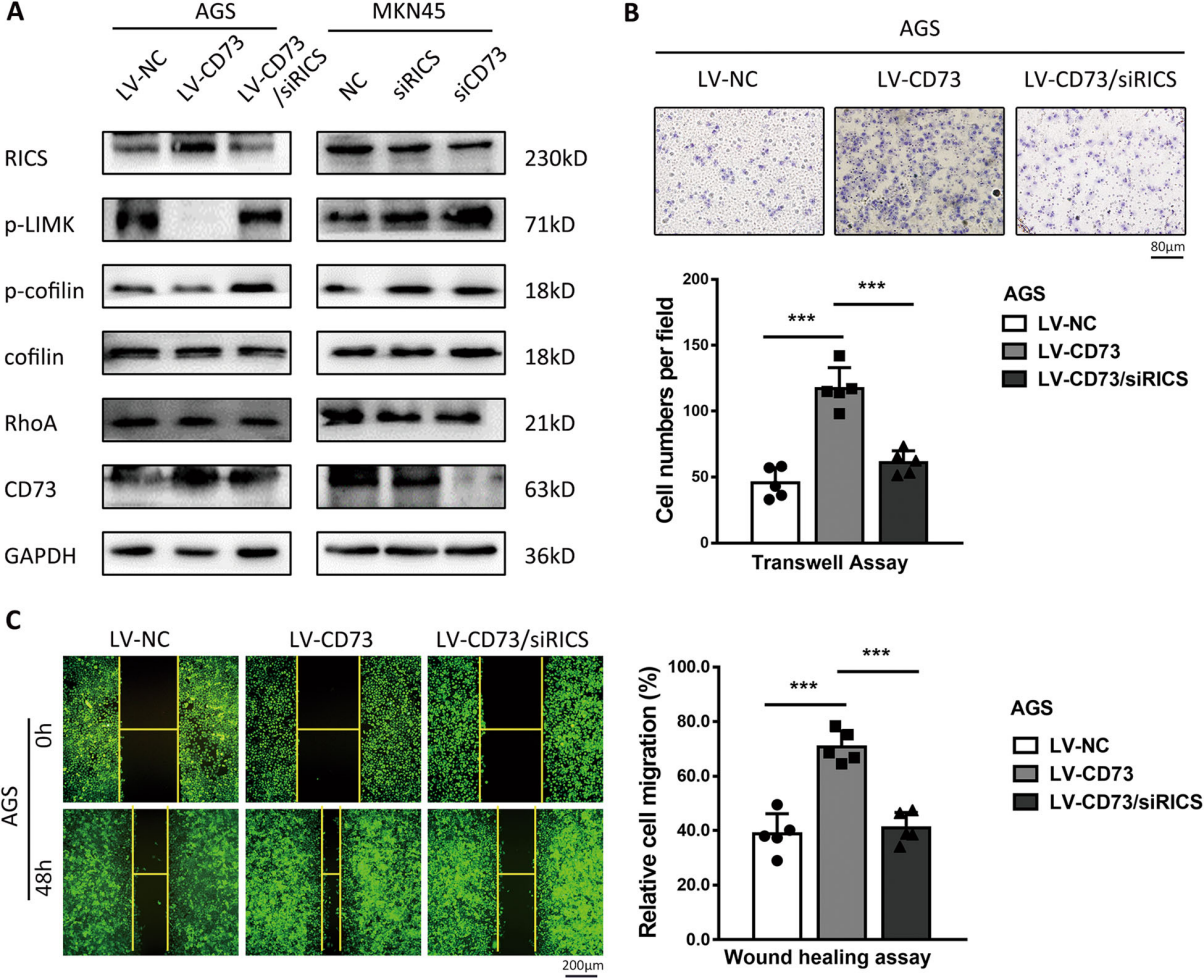
RICS is essential for cytoskeletal regulation and invasive properties by CD73. **a** Western blot analysis of RICS, phosphorylation level of RhoA-LIMK-cofilin signaling and CD73 in AGS-LV-CD73 or MKN45 cells transfected with siRICS or siCD73 as indicated. Depletion of RICS in AGS-LV-CD73 cell was found to rescue phosphorylated LIMK and cofilin induced by overexpression of CD73. **b** Migration assessment via transwell assay in AGS-LV-CD73 cell transfected with siRICS (mean ± SD, *n* = 5, one-way ANOVA). **c** Wound healing assay detecting RICS function in CD73-induced GC migration (mean ± SD, *n* = 5, one-way ANOVA). ***p < 0.001.

RICS was predicted to interact with multiple cytoskeletal proteins by protein–protein interaction network, and among them, RhoA was a direct substrate of RICS (Supplementary Fig. 1D). WB detection in CD73-overexpressed GC cells confirmed that phosphorylation of LIM kinase (p-LIMK) and cofilin (p-cofilin), direct substrates of GTP-bound RhoA, were reduced by activation of RICS, indicating suppression effects of RICS in RhoA activity (Fig. 4d). GTPase-activating activity (GAP) of RICS toward RhoA abolished the phosphorylation process of RhoA on LIMK1, LIMK2 by phosphorylation of Thr508 and Thr505, respectively, and subsequently the phosphorylation of cofilin by LIMK^24^. Accordingly, depletion of CD73 in MKN45 cells decreased the expression of RICS and eventually promoted the phosphorylation of LIMK and cofilin (Fig. 4d). To further determine whether RICS function was essential for cytoskeletal regulation and invasion properties induced by CD73, we thus transfected RICS siRNA into AGS-LV-CD73 cell and found rescued expression of p-LIMK and p-cofilin (Fig. 5a). Similarly, inhibition of RICS in MKN45 cells could induce elevated phosphorylation of LIMK and cofilin, indicating the role of RICS as an intermediate signaling component in cytoskeletal regulation pathway (Fig. 5a). Consistent with the effect of RICS in the modulation of RhoA/LIMK/cofilin signaling, migration ability of GC cells was partly abolished by RICS interference in CD73-overexpressed GC cells, as shown in transwell and wound healing assay (Fig. 5b, c). Taken together, these findings highlighted that CD73/RICS inhibited the phosphorylation of RhoA-LIMK-cofilin signaling and enhanced EMT process in GC cells, and ultimately affected the cytoskeletal rigidity and promoted more invasive phenotype of GC.

### Pharmacological inhibition of CD73 adenosinergic signaling induces RICS dysfunction

**Fig. 6:**
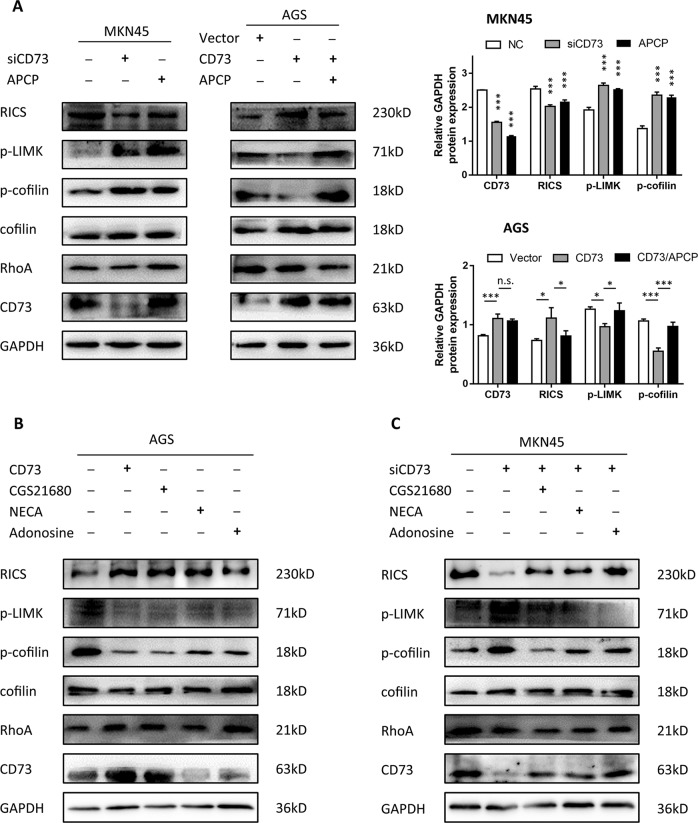
Pharmacological inhibition of CD73 adenosinergic signaling induces RICS dysfunction. **a** WB analysis of phosphorylation level of RhoA/LIMK/coflin signaling in MKN45 cells treated with APCP or siCD73, and AGS cells transfected with CD73 plasmids. Relative gray value was shown (mean ± SD, *n* = 3, one-way ANOVA). **b** WB analysis of inhibition effects of CD73 plasmids, adenosine receptor agonist (CGS21680, NECA), or adenosine in RhoA/LIMK/cofilin signaling. **C** WB analysis of phosphorylation level of RhoA/LIMK/coflin signaling to determine rescued function of adenosine receptor agonist or adenosine in MKN45 cells transfected with siCD73. *p < 0.05, ***p < 0.001, and N.S., non-significant.

Adenosine production and adenosinergic signaling were defined to link enzymatic activity of CD73 switching to intracellular signaling through activation of multiple adenosine receptors (AR, including A1, A2A, A2B, and A3) distributed on cell membranes. We thus explored whether pharmacological blockage or stimulation of adenosinergic signaling could induce alteration of RICS function in GC. First, APCP treatment was used to decrease AMPase activity of CD73 and it exhibited nearly unanimous activation effects in RhoA/LIMK/coflin signaling compared with CD73-knockdown group (Fig. 6a). Meanwhile, APCP treatment abolished the CD73-induced suppression in p-LIMK and p-cofilin in CD73-overexpressed cells, indicating that CD73 regulated RICS function via enzyme-dependent activity (Fig. 6a). To further confirm adenosine-facilitated activation of AR was a key for modulating RICS function, adenosine was employed and found to promote RICS-induced suppression of RhoA/LIMK/cofilin signaling in GC cells (Fig. 6b). Additionally, CD73-silenced GC cells also showed rescued modulation effects in RICS function when exposed to adenosine treatment (Fig. 6c). Collectively, it showed a vital role of activated adenosinergic pathway involved in CD73-induced RICS function.

We next search for the exact AR involved in the activation of intracellular signaling in GC using selective A2AR agonist CGS21680 and non-selective AR agonist NECA. As a result, both CGS21680 and NECA inhibited the phosphorylation of RhoA-LIMK-cofilin signaling, but NECA showed the reduction of p-LIMK and p-cofilin to the maximum extent in AGS cells (Fig. 6b). We confirmed this finding by adding CGS21680 and NECA as restoration of adenosinergic signaling in CD73-knockdown MKN45 cells, and similar effects were observed. CGS21680 partially rescued the inhibition effects of CD73, while NECA dramatically suppressed the expression of p-LIMK and p-cofilin (Fig. 6c). Together, it revealed that both A2AR agonist and non-selective AR agonist could activate RICS modulation in RhoA/LIMK/cofilin signaling and CD73 was confirmed to modulate RICS function via almost all ARs. Pharmacological inhibition of non-selective AR might abolish the CD73 intracellular function to a greater extent.

### Targeting CD73 suppresses GC metastasis in vivo

**Fig. 7:**
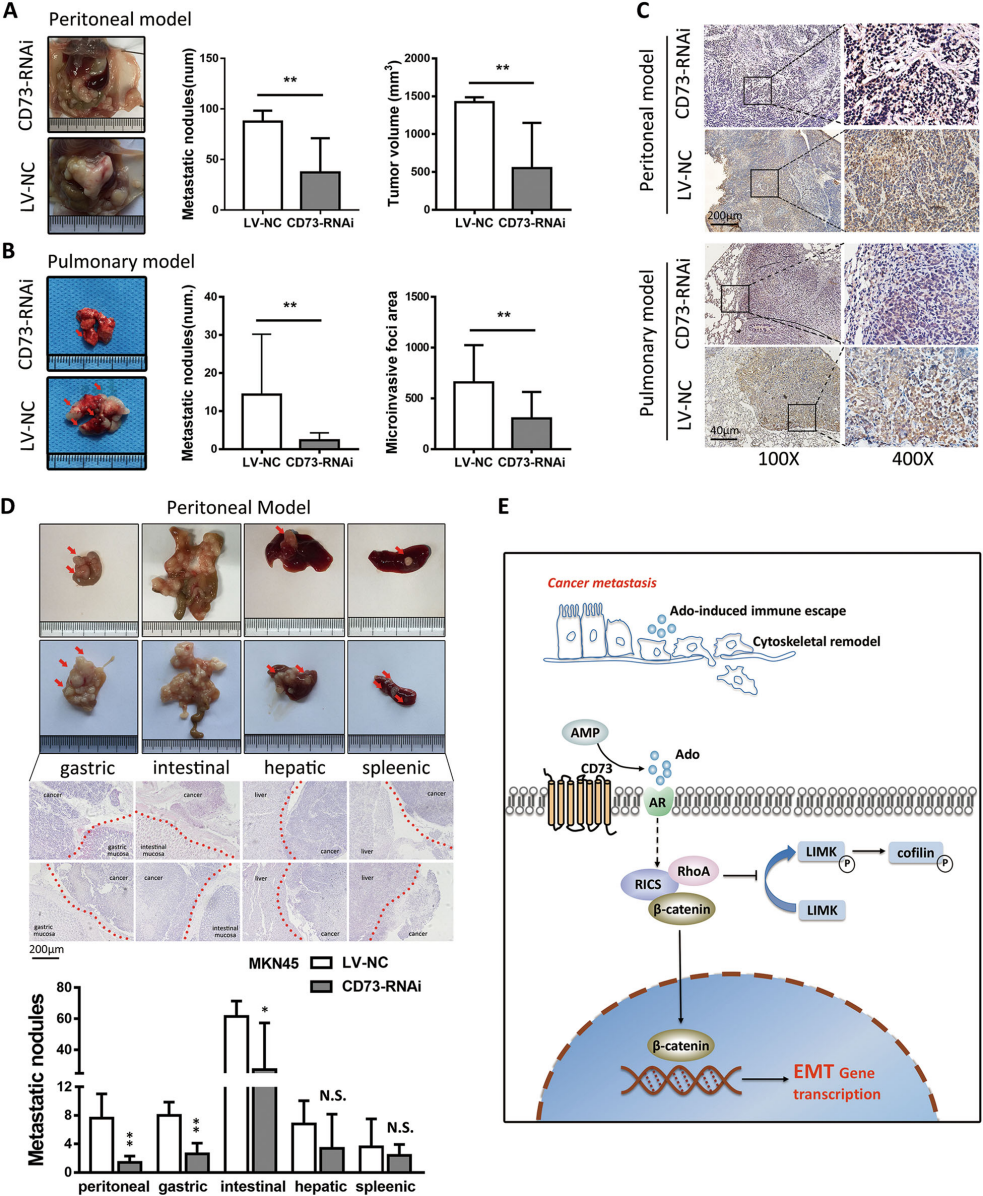
Genetic depletion of CD73 inhibits tumor metastasis of GC in vivo. **a** White-light images of peritoneal metastasis in the seeding metastasis model using MKN45 cancer cells (LV-NC, CD73-RNAi). Number of metastasis nodules and tumor volume were analyzed (mean ± SD, *n* = 5, unpaired *t* test). **b** White-light images of pulmonary metastasis in hematogenous metastasis model using MKN45 cancer cells and nodules were indicated with red arrow. The number of metastasis nodules and microinvasive area were analyzed (mean ± SD, *n* = 6, unpaired *t* test). **c** IHC staining of CD73 in metastasis nodules in seeding metastasis model and hematogenous metastasis model. **d** White-light images of gastric, intestinal, hepatic, and splenic tumor nodules and HE staining of metastasis nodules were shown. Red arrow indicates the nodules and red imaginary line indicates the border line of implanting sites (mean ± SD, *n* = 5, unpaired *t* test). **e** Diagram summarizing the role of CD73 in modulating metastasis process of GC. CD73-induced adenosinergic signaling modulated RICS function in the phosphorylation of RhoA-LIMK-cofilin signaling and activation of β-catenin-induced EMT process. *p < 0.05, **p < 0.01, and N.S., non-significant.

As pharmacological inhibition might limit the suppression effects in function of CD73, we used genetic inhibition to investigate the treatment effects of targeting CD73 in the metastasis process of GC. Human GC cells expressing either scramble or CD73 short hairpin (shRNA) were inoculated into peritoneal cavity or tail vein to generate peritoneal seeding and hematogenous metastasis model. Results showed that in both two models, LV-NC group showed significantly more metastatic nodules and greater size of tumor (Fig. 7a–c). In hematogenous metastasis model, genetic inhibition of CD73 reduced the homing capacity of GC cells and dissemination area (Fig. 7b). Subgroup analysis of metastasis location in peritoneal metastasis model revealed that tumor nodules and volume significantly decreased mainly in peritoneum, gastric, and colorectal metastasis sites, but not in hepatic and splenic sites (Fig. 7d). Collectively, targeting CD73 suppressed the dissemination and colonization of GC cells in vivo.

## Discussion

Growing evidence shows that TME formation and oncogenic mutations are hallmarks of cancer^25^. Specific oncogenes in TME link intrinsic properties of cancer cells switching to modulation of TME and eventually induce unbridled growth or metastasis of cancer^26–28^. Recently, targeting biochemical composition in TME has become a frontier of cancer therapy, and among them, adenosinergic signaling was found to operate in the milieu of most cancer^29^. However, its role and mechanism in GC progression and metastasis remains largely unknown. Here we reported that CD73, modulator of TME adenosine production via a catalysis way, was overexpressed in GC and acted as a driver gene in inducing metastasis process. Functional and mechanistic studies suggested that CD73 triggering adenosinergic signaling acted as a regulator of cytoskeleton by crosstalk with intracellular RICS, which inhibited phosphorylation of RhoA-LIMK-cofilin pathway and activated β-catenin-induced EMT process via a direct interaction, ultimately resulting in metastasis property of GC (Fig. 7e). Pharmacological inhibition of CD73 confirmed the effects of adenosinergic signaling blockage in inducing RICS dysfunction, and multiple ARs were verified to involve in CD73 intracellular function. Targeting CD73 shows a more available prevention of GC metastasis in vivo.

Accruing evidence over the past few years shows that CD73 is involved in metastasis of various kinds of cancer, dependent on its enzyme activity in production of adenosine in TME^17,30–32^. It has been demonstrated that elevated concentration of extracellular adenosine, which was always elevated by oxidative stress, correlated with immune and inflammation response via activation of immunosuppressive Tregs or inhibition of antitumor immune cells^33^. However, recent advance in adenosinergic signaling reveals its plentiful function beyond the immune system. In villus tip enterocytes and vasculature, adenosine derived from AMP by CD73 and activation of adenosine receptors were defined to protect epithelial integrity and barrier function, indicating crosstalk between adenosinergic signaling and actin dynamics^34,35^. Our data supported the concept that CD73-induced adenosinergic signaling promotes cytoskeleton regulation and cancer metastasis in GC. Acquisition of more flexible motility via cytoskeletal rearrangement had been regarded as prerequisites for cancer metastasis or occurrence. A group of ARHGAPs have been characterized to facilitate actin polymerization and cancer cell morphology via GAP activity in Rho GTPases, including Rho, Rac, and Cdc42 subfamilies, and is responsible for modulation of pivotal pathways during the cancer metastasis like p53, Hippo, and PKA signaling^24^. Here we affirmed the enrichment of cytoskeletal regulation by Rho GTPase pathway induced by CD73 and defined RICS, a Rho GTPase-activating protein, as the “bridge” molecule. Consistent with our results, previous studies in purinergic signaling also suggested the mechanotransducer role of different kinds of purinergic receptor in actin dynamics or cytoskeletal disorganization^36–38^.

RICS was originally regarded as an enriched GAP protein to promote hydrolysis of GTP-bound RhoA, CDC42, and Rac1 in postsynaptic density^39^. Only GTP-bound RhoA can activate Rho-associated kinase, which then phosphorylated LIMK1 and LIMK2 by Thr508 and Thr505, respectively, resulting in the phosphorylation of cofilin and stabilization of actin filaments^40^. Our finding suggested that RICS was a downstream target of CD73-induced adenosine pathway and acted as a destroyer of cytoskeleton stabilization via inhibiting phosphorylation of LIMK and cofilin, which partly explained its promotion in cell invasion and migration. LIMK contains two distinct protein kinases (LIMK1, LIMK2) and both of them had been verified to be involved in cancer progression and metastasis^41–43^. According to recent research, imbalance of these two highly related members of LIMK family resulted in colorectal cancer progression and metastasis via promoting β-catenin nuclear translocation^41,44^. We have identified the similar phenomenon induced by CD73 via adenosine pathway in this study. Precedent works showed that RICS could directly interact with β-catenin in vivo and the RICS–β-catenin complex is associated with E-cadherin and N-cadherin^23^. We supposed the direct relationship between RICS and β-catenin in GC, and from another perspective, RICS might induce β-catenin activation via an indirect function of LIMK. However, it still calls for more evidence to confirm the hypothesis.

Pharmacological inhibitors of adenosinergic signaling in TME were well investigated for their functions in immunosuppression and linking membrane proteins like CD73 with intracellular signaling modulation^12,45,46^. Some of them showed significant therapeutic effects in mouse model or preclinical trials^45,47–49^. We explored potential receptor assuming intracellular function of CD73 and identified A2AR as a critical receptor in modulating RICS function. However, we also found that targeting AR had limited effects in blocking CD73 function, for its broader impression in multiple ARs. Previous studies had showed similar function and crosstalk among diverse ARs in TME, which could result in failure of targeting special AR in cancer therapy. Thus, targeting CD73 by genetic inhibition or monoclonal antibodies (mAbs) might be a preferred way. Here we provided strong evidence that targeting CD73 in primary tumor could dramatically abolish the effects of CD73 and eventually suppress experimental metastasis in both peritoneal seeding and hematogenous metastasis model. Accumulating evidence had revealed the antitumor effects by targeting CD73 in immune microenvironment, which resulted in proliferation and effector functions of cytotoxic lymphocytes while simultaneously promoting the generation and infiltration of immunosuppressive cells^50^. In addition, recent studies have revealed an enhanced antitumor effects of anti-PD-1 or anti-CTLA-4 mAb in mouse model when combined with anti-CD73 mAb^51^. We demonstrated a similar conclusion in this study, and more importantly, targeting CD73 was found to weaken the intrinsic metastasis properties of GC.

In conclusion, our study identified overexpression of CD73 and its involvement in GC metastasis via the adenosinergic signaling-dependent regulation of RICS and RhoA-LIMK-cofilin signaling, which could be blocked with pharmacological inhibition of AR. CD73 depletion was effective in reducing cancer metastasis in xenograft nude mice, indicating CD73 as a therapeutic target for GC metastasis.

## Materials and methods

### Patients and specimens

Fresh primary GC specimens and paired normal gastric mucosal tissues were collected from patients who had received routine surgery for GC and volunteered to provide samples for research in Nanfang Hospital of Southern Medical University (Guangzhou, China). All the subjects gave written informed consent about the acquirement of samples and privacy protection. Among them, GC tissues from a cohort containing 171 cases of consecutive patients with complete clinical and pathological data, and follow-up time for more than 3 years were used for the manufacture of TMA by Servicebio company (Wuhan, China). Collection of human tissues and analysis of clinical data were approved by the Ethics Committee of Nanfang Hospital.

### Cell lines and cell culture

Human gastric cancer cell lines MKN45, SGC7901, AGS, MGC803, and BGC823 and human gastric mucosal cell line GES-1 were obtained from American Type Culture Collection (ATCC, Manassas, VA, USA), generated in the Department of General Surgery, Nanfang Hospital, and authenticated by short tandem repeat (STR) profiling and tested for mycoplasma contamination. Cell lines were cultured in RPMI-1640 medium (BI) with 10% fetal bovine serum (FBS) (Gibco) at 37 °C with 5% CO_2_.

### RNA isolation, qPCR, and WB

Total RNA from samples or cultured cells was isolated with Trizol reagent (TakaRa) and qPCR was performed with PrimeScript RT Reagent Kit (TakaRa) and SYBR Premix Ex Taq (TakaRa) according to the manufacturer’s instructions. Primers are listed in Supplementary Table 2 and GAPDH (glyceraldehyde 3-phosphate dehydrogenase) was used as an internal control. Standard WB procedure was performed and protein was transferred to nitrocellulose membrane (Bio-Rad). Five percent of nonfat milk was used for blocking and the membranes were incubated with special primary antibodies and visualized with Immobilon ECL (Millipore).

### HE analysis and IHC

Standard procedure of hematoxylin–eosin (HE) analysis and IHC staining were performed to evaluate the expression level of proteins as previously described^52^. Tumor samples were obtained from nude mice or patients receiving surgery in Nanfang Hospital. Sections for IHC were incubated with CD73 (Abcam, 1:300) and intensity of staining of cancer cells was scored as: 0 (no staining), 1 (weakly staining, light yellow), 2 (moderately staining, yellowish brown), and 3 (strongly staining, brown). Evaluation was analyzed by three independent observers using the same light microscope. An average intensity score of ≥2 was considered as overexpression, whereas <2 in the intensity score was regarded as low expression.

### IF assays

Cells were fixed in a 4% paraformaldehyde solution and added a 1% Triton solution to penetrate the cytomembrane. After incubating with antibodies (CD73, Abcam, ab81720 1:50, E-cadherin, Proteintech, 20874-1-AP 1:50, β-catenin, Proteintech, 17565-1-AP 1:50, vimentin, Proteintech, 10366-1-AP, 1:50) overnight at 4 °C, a fluorescent secondary antibody and the DAPI Staining Kit were used for observation of primary antibody and cell nuclear, respectively.

### Plasmids, siRNA transfection, and lentiviral infection

siRNA sequences targeting CD73 and RICS are shown in Supplementary Table 2. siRNA pools were used to transfect GC cells using Lipofectamine 3000 (Invitrogen). siRNA of CD73 was chemically synthesized by GeneChem (Shanghai, China) and introduced into the GV248 lentiviral vector using the *Age*I/*Eco*RI sites. To construct human CD73 expression plasmid, the DNA fragment of human CD73 was subcloned into the GV358 lentiviral vector using the *Age*I/*Age*I sites. The GV248, GV358 vectors both contain the enhanced green fluorescent protein (EGFP) coding sequence and a IRES-puromycin cassette. Constructed vectors were transfected into lentiviral packaging cell lines HEK293T, and lentiviral particles were collected from the supernatant of the transfected cells. MKN45 and AGS cells (1 × 10^5^) were infected with 1 × 10^7^ lentivirus, and after 72 h of transfection, efficacy of infection was evaluated according to EGFP expression in the fluorescence microscope.

### Reagents

SP600125 (20 μM, Millipore) as an inhibitor of phosphorylation of c-Jun was first dissolved in dimethyl sulfoxide and added to cultured cells for 48 h. CGS21680 (100 nM, MCE), NECA (10 μM, MCE), adenosine (10 μM, MCE) as an activator, and APCP (100 μM, Sigma) as an inhibitor in adenosine pathway were used for treatment in cell lines.

### Wound healing and migration assay

Approximately 5 × 10^5^ cells per well were seeded into 6-well plates and five parallel scratches or “wounds” (wide approximately equal to 500 μm) were marked after the cells becoming adherent. Migration of cells into the “wounds” was observed and images of areas flanking the intersections of the “wound” and the marked lines were taken at regular intervals over the course of 24 h. For migration assay, cells were harvested and resuspended in serum-free RPMI-1640 medium, and 5 × 10^4^ cells were placed into 6.5-mm Boyden chambers with 8-μm pores (Corning Costar, Corning, NY, USA) and then inserted into the wells of a 24-well plate and incubated for 24 h in RPMI-1640 medium with 10% FBS prior to examination. Cells adhering to the lower surface were fixed and stained in a dye solution containing 0.05% crystal violet and counted under the microscope to determine their relative numbers. For each experiment, the number of cells in at least five random field on the underside of the filter was counted, and three independent filters were analyzed.

### In vivo metastasis assay

Animals were allocated to the experimental and control group according to a random number table and no blinding was done. Five-week-old male BALB/c nude mice were purchased from the Laboratory Animal Services Center of Guangdong Province and maintained at the Laboratory Animal Center of Nanfang hospital in a specific pathogen-free environment. For the construction of seeding metastasis model, 5 × 10^6^ MKN45 cells (LV-NC, CD73-RNAi) were injected into the peritoneal cavity of nude mice (*n* = 5 per group) and the mice were euthanized and all organs were removed for examination 6 weeks later. Peritoneal, gastric, intestinal, hepatic, and splenic metastases were detected by HE and IHC staining and quantified by counting metastatic lesions in each section. To evaluate the hematogenous metastasis capacity, a total of 5 × 10^5^ MKN45 cells were injected via the tail veins of mice (*n* = 6 per group) and mice were euthanized for examination 6 weeks later. Nude mice experiments were approved by the ethics committee of the Nanfang Hospital, Southern Medical University.

### Bioinformatic analysis

Expression profiling data of mRNA analyzed in this study were downloaded from TCGA (http://cancergenome.nih.gov/). GSEA analysis was performed as previously described (http://software.broadinstitute.org/gsea/index.jsp) and normalized enrichment score (NES) and *p* value have been shown^53^. KOBAS 3.0 (http://kobas.cbi.pku.edu.cn/index.php) was used for pathway enrichment analysis of differently expressing genes (fold change ≥1.5) according to PANTHER pathway public datasets^54^. Correlation between CD73 and RICS was analyzed with stomach adenocarcinoma datasets from TCGA via gene expression profiling interactive analysis^55^.

### Statistical analysis

Differences were considered significant if *p* < 0.05. Sample size was chosen according to previous observations, which perform similar experiments to see significant results, or the results from our preliminary experiments. Thus, experiments including WB blot, QPCR, transwell, and wound healing assay were repeated three times in this study. For animal studies, sample size was estimated to be no <5 in seeding or hematogenous metastasis models. For CD73 detection in human samples, sample size was estimated to be >30 as a large sample. GraphPad Prism software (Version 5.0, GraphPad Software Inc., San Diego, CA, USA) and SPSS software (Version 19.0; Abbott Laboratories, Chicago, IL) were used for statistical analysis. Data were described as mean ± standard error of mean or proportion unless otherwise noted. The Student’s *t* test was used to detect significance between groups and *χ*^2^ test was used for measurement data. ANOVA (analysis of variance) analysis was used for analysis of differences among three or more groups and post-hoc analysis was performed. Multiple comparisons were done after homogeneity test for variance. Variance was similar between the groups that are being statistically compared. For correlation of IHC grades and AJCC stage, linear-by-linear association analysis was used. Correlation between CD73 and RICS with standardized mRNA data from TCGA was analyzed with Pearson’s correlation. The effects of multiple variables on survival were determined by univariate and multivariate Cox proportional hazards modeling. Kaplan–Meier and log-rank tests were then used for survival curves analysis.

## Supplementary information

Supplementary Figure 1

Supplementary Figure 2

Supplementary Figure 3

Supplementary Figure 4

Supplementary figure legends

Supplementary Table 1

Supplementary Table 2

Supplementary Table 3

Reproducibility Checklist

Supplementary file cddis-author-contribution-form.pdf
