# Supplementary figures and images for: CD73 promotes tumor metastasis by modulating RICS/RhoA signaling and EMT in gastric cancer
Source: Cell Death Dis. 2020 Mar 23;11(3):202. doi: 10.1038/s41419-020-2403-6 (PMC7089986; doi:10.1038/s41419-020-2403-6)

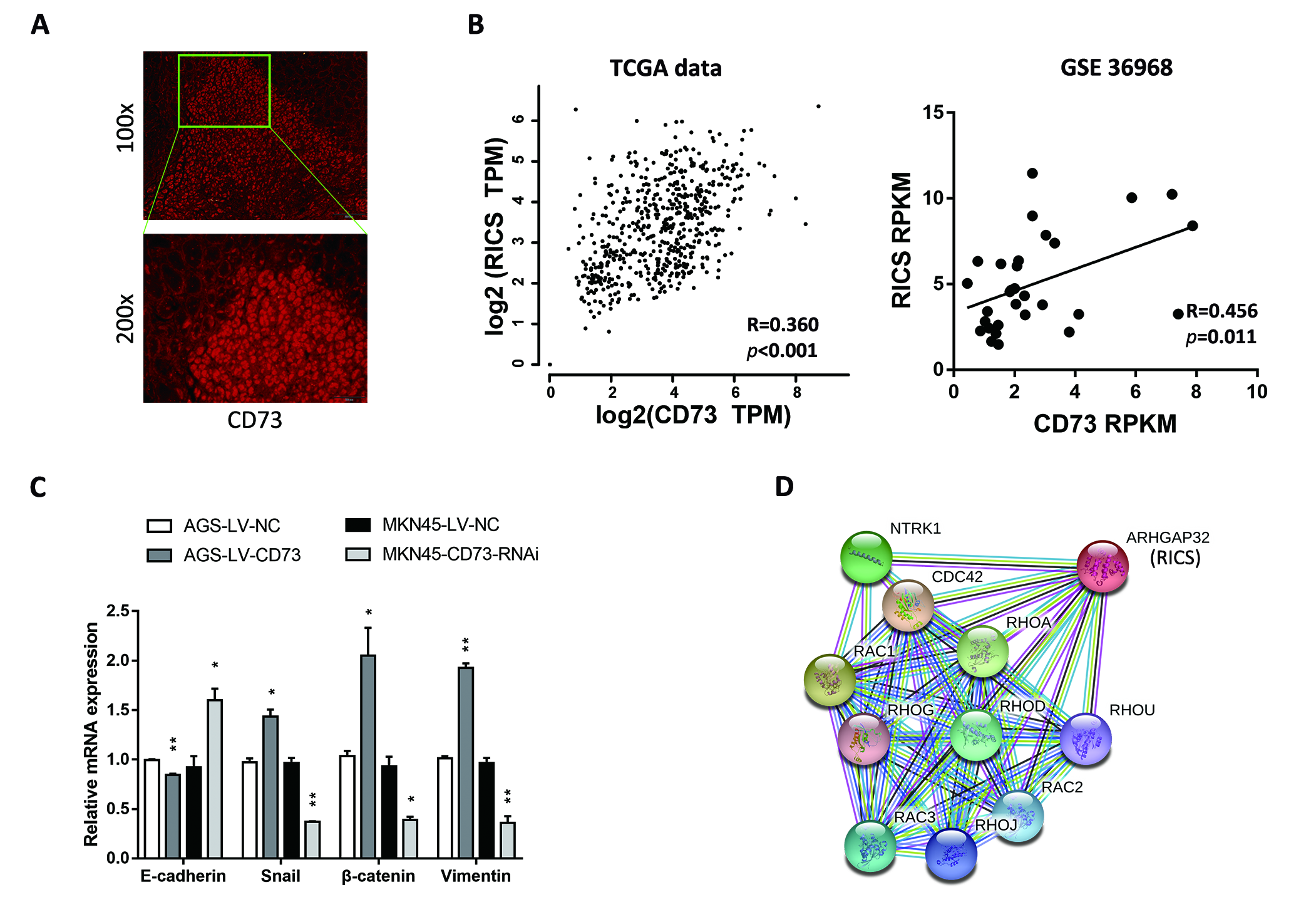

Supplement: Supplementary file 1 — Supplementary Figure 1 [file 41419_2020_2403_MOESM1_ESM.tif]

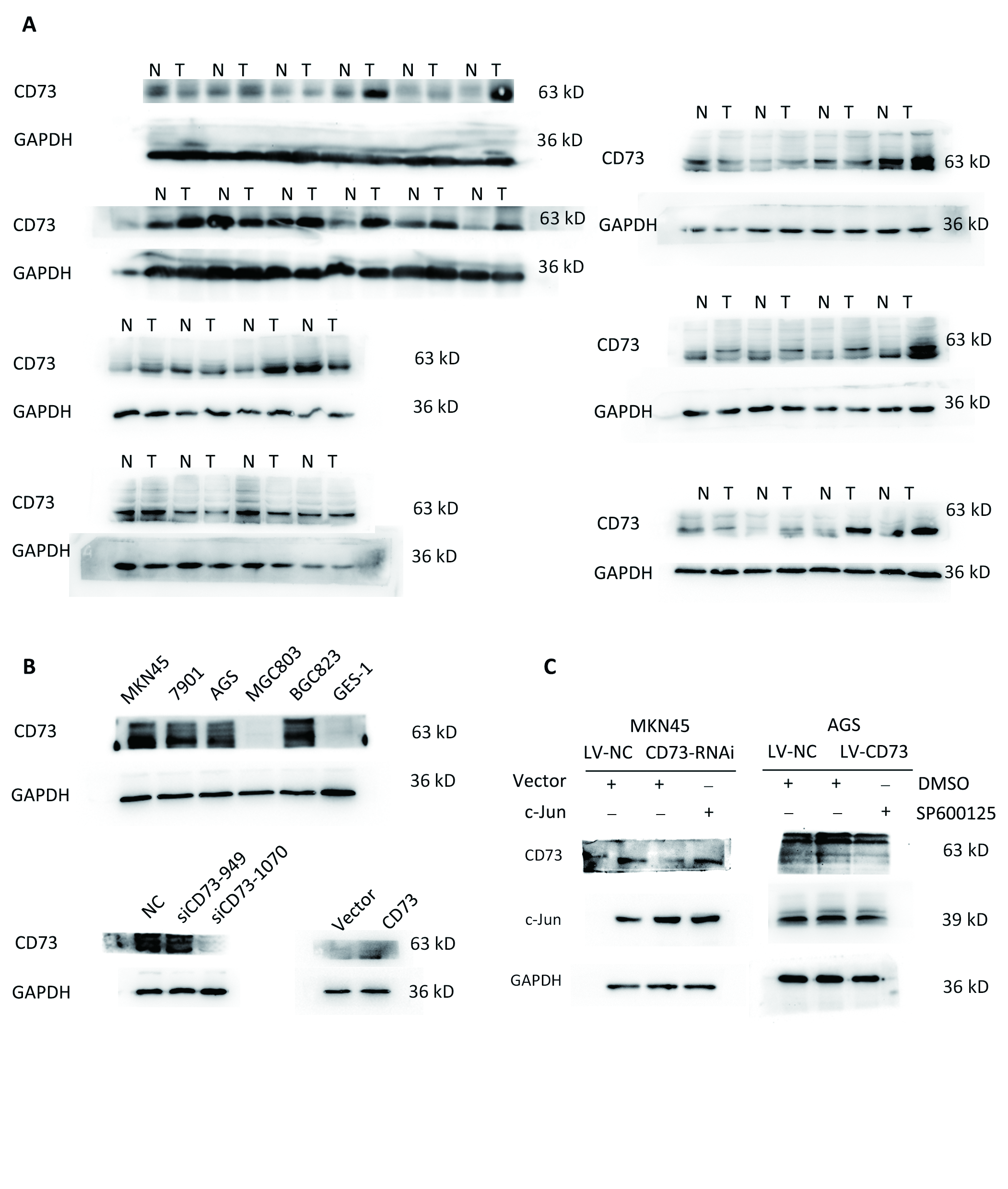

Supplement: Supplementary file 2 — Supplementary Figure 2 [file 41419_2020_2403_MOESM2_ESM.tif]

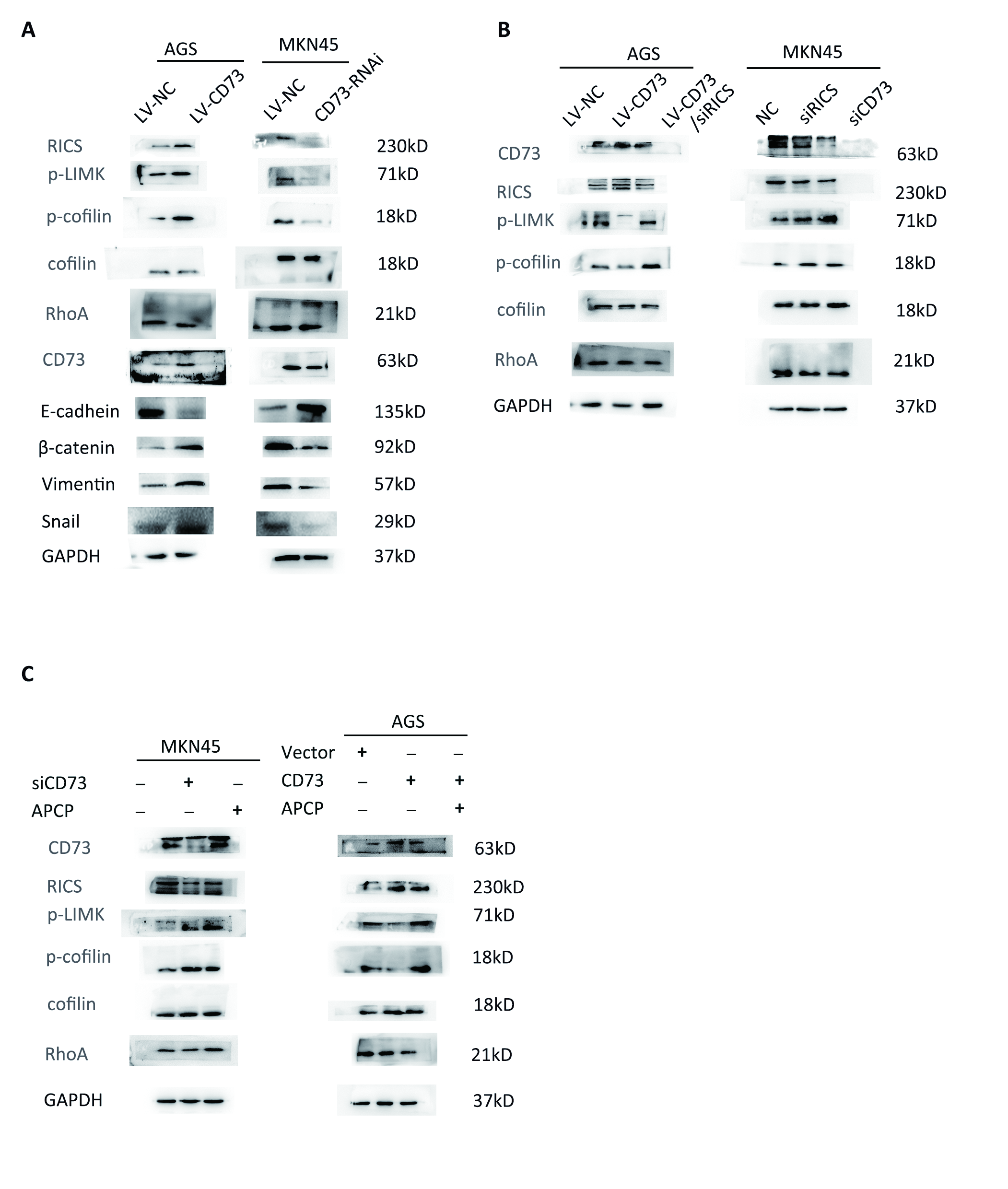

Supplement: Supplementary file 3 — Supplementary Figure 3 [file 41419_2020_2403_MOESM3_ESM.tif]

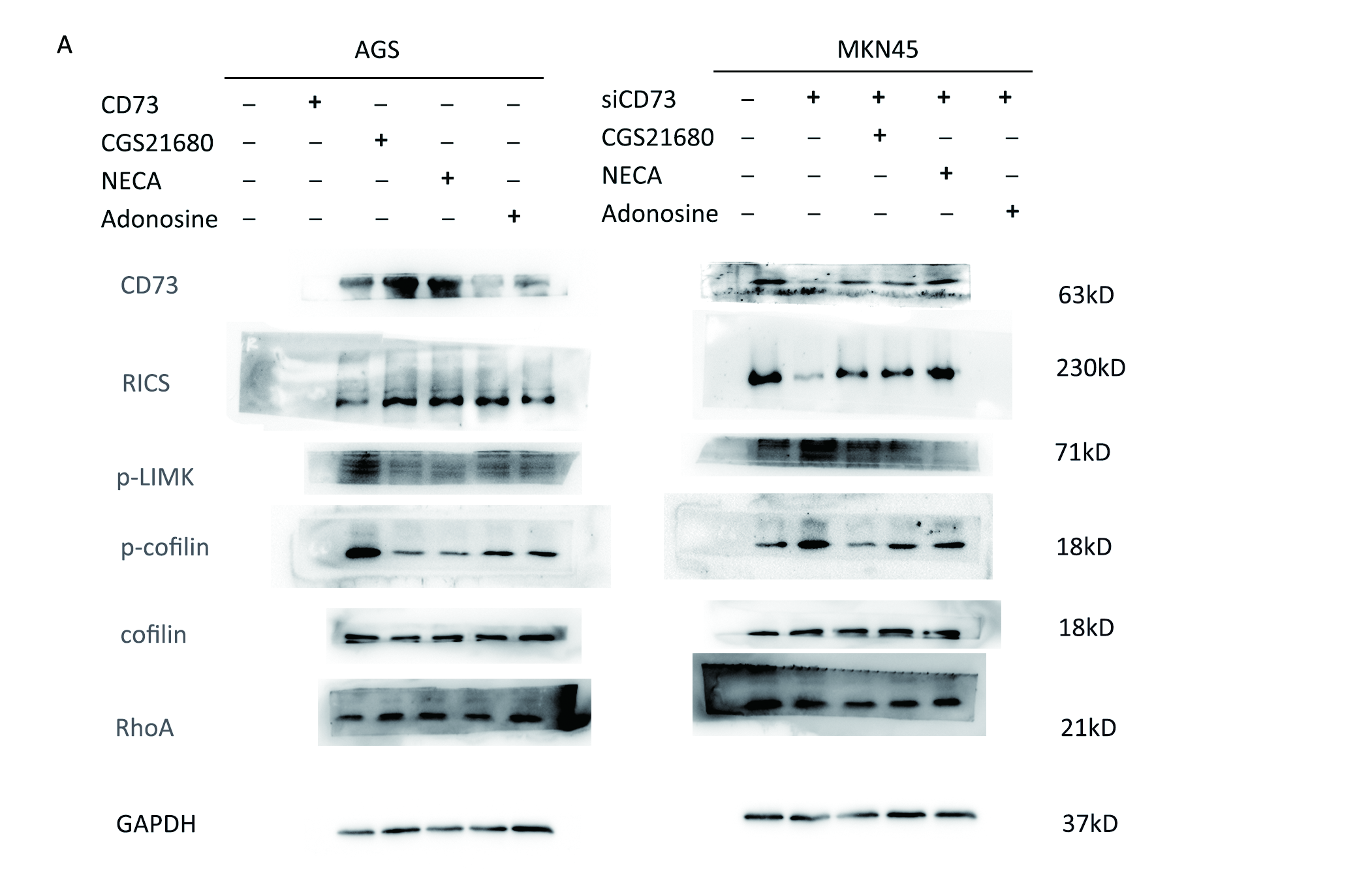

Supplement: Supplementary file 4 — Supplementary Figure 4 [file 41419_2020_2403_MOESM4_ESM.tif]
