# Supplementary material for: CD73 promotes tumor metastasis by modulating RICS/RhoA signaling and EMT in gastric cancer
Source: Cell Death Dis. 2020 Mar 23;11(3):202. doi: 10.1038/s41419-020-2403-6 (PMC7089986; doi:10.1038/s41419-020-2403-6)
Supplement: Supplementary file 5 — Supplementary figure legends [file 41419_2020_2403_MOESM5_ESM.doc]

**Supplementary Figure 1** Expression and functional relation between CD73 and RICS. **A** IF staining of CD73 in boarder line of GC. **B** Correlation between CD73 and RICS according to TCGA and GSE36968 expression data. **C** Relative mRNA levels of EMT-associated proteins in AGS-LV-NC, AGS-LV-CD73, MKN45-LV-NC, MKN45-CD73-RNAi cells. **D** Protein-protein interaction between RICS and cytoskeleton proteins.

**Supplementary Figure 2** Uncropped whole blot images of western blot data. **A** WB data in Figure1A**. B** WB data in Figure2A. **C** WB data in Figure3A,B.

**Supplementary Figure 3** Uncropped whole blot images of western blot data. **A** WB data in Figure4D**. B** WB data in Figure5A. **C** WB data in Figure6A.

**Supplementary Figure 4** Uncropped whole blot images of western blot data. **A** WB data in Figure6B**. B** WB data in Figure6C.
