# Supplementary material for: CD73 promotes tumor metastasis by modulating RICS/RhoA signaling and EMT in gastric cancer
Source: Cell Death Dis. 2020 Mar 23;11(3):202. doi: 10.1038/s41419-020-2403-6 (PMC7089986; doi:10.1038/s41419-020-2403-6)
Supplement: Supplementary file 6 — Supplementary Table 1 [file 41419_2020_2403_MOESM6_ESM.docx]

Supplementary Table 1 Univariate and Multivariate Analysis of Different Prognostic Parameters for GC Patients.

| Variables | Univariate | |  | Multivariate | |
| --- | --- | --- | --- | --- | --- |
|  | HR (95% CI) ^a^ | *p* Value ^b^ |  | HR (95% CI) ^a^ | *p* Value ^b^ |
| Age (>65 vs. ≤65) | 1.004(0.386,2.613) | 0.993 |  |  |  |
| BMI (>30 vs. ≤30) ^c^ | 2.024(0.701,5.849) | 0.193 |  |  |  |
| ECOG (>1 vs. ≤1) ^d^ | 0.602(0.301,1.203) | 0.151 |  |  |  |
| Gender(Male vs Female) | 0.362(0.208,0.631) | <0.001 |  | 0.468(0.279,0.786) | 0.004 |
| Lesion(single vs multiple) | 2.115(0.352,12.693) | 0.413 |  |  |  |
| Tumor size (<5.0cm vs ≥5.0cm) | 2.691(1.224,5.916) | 0.014 |  | 1.936(1.01,3.713) | 0.047 |
| Surgery (standard vs non-standard) | 2.272(1.215,4.248) | 0.010 |  | 2.288(1.307,4.006) | 0.004 |
| Bormann’s classification(I/II vs III/ IV) | 0.837(0.415,1.688) | 0.618 |  |  |  |
| Resection margin (positive vs negative) | 2.618(0.503,13.636) | 0.253 |  |  |  |
| L/V/N infiltration (positive vs negative) ^e^ | 0.748(0.288,1.938) | 0.549 |  |  |  |
| AJCC stage (I/II vs III/IV) | 21.307(6.894,65.858) | <0.001 |  | 17.504(6.258,48.961) | <0.001 |
| CD73 expression(Low vs High) | 2.161(1.177,3.968) | 0.013 |  | 1.921(1.096,3.367) | 0.023 |

^a^ Hazard ratios (HRs) and 95% confidence intervals (CIs) were calculated using univariate or multivariate Cox proportional hazards regression in SPSS.

^b^ *p* values were calculated using univariate or multivariate Cox proportional hazards regression in SPSS. *p* values < 0.05 were considered to indicate statistical significance.

^c^ BMI, body mass index

^d^ ECOG, Eastern Cooperative Oncology Group performance status

^e^ L, lymphatic; V, vascular; N, perineural
