# Supplementary material for: CD73 promotes tumor metastasis by modulating RICS/RhoA signaling and EMT in gastric cancer
Source: Cell Death Dis. 2020 Mar 23;11(3):202. doi: 10.1038/s41419-020-2403-6 (PMC7089986; doi:10.1038/s41419-020-2403-6)
Supplement: Supplementary file 7 — Supplementary Table 2 [file 41419_2020_2403_MOESM7_ESM.doc]

**Supplementary Table 2**

Sequence information used in this study

| **siRNA** | **Sense** | **antisense** |
| --- | --- | --- |
| **siCD73-949** | 5'-CCUCAAAGAGGCCAAAUUUTT-3' | 5'-AAAUUUGGCCUCUUUGAGGTT-3' |
| **siCD73-1070** | 5'-GGAAUCGUUGGAUACACUUTT-3' | 5'-AAGUG UAUCC AACGA UUCCTT-3' |
| **siRICS-1564** | 5'-GCAAUUGUUUGGGCUCCAATT-3' | 5'-UUGGAGCCCAAACAAUUGCTT-3' |
| **siRICS-1752** | 5'-GCCCAAGUCCCUCCUGGUATT-3' | 5'-UACCAGGAGGGACUUGGGCTT-3' |
| **siRICS-3672** | 5'-CCAUCACCCUUUAGAAUUUTT-3' | 5'-AAAUUCUAAAGGGUGAUGGTT-3' |
| **Gene** | **Forward** | **Reverse** |
| **CD73** | 5'-TCGGGTTTTGAAATGGATAAAC-3' | 5'-TCAGGAATGCTGCTGTTTAGAA-3' |
| **GAPDH** | 5'-GGAGCGAGATCCCTCCAAAAT-3' | 5'-GGCTGTTGTCATACTTCTCATGG-3' |
