# Supplementary material for: CD73 promotes tumor metastasis by modulating RICS/RhoA signaling and EMT in gastric cancer
Source: Cell Death Dis. 2020 Mar 23;11(3):202. doi: 10.1038/s41419-020-2403-6 (PMC7089986; doi:10.1038/s41419-020-2403-6)
Supplement: Supplementary file 8 — Supplementary Table 3 [file 41419_2020_2403_MOESM8_ESM.doc]

**Supplementary Table 3** Raw data for statistical analyses

**Table S3-1 Relative RNA expression level of CD73 in 32 pairs of GC tissues**

| 1 | 1.000974 | 2.512569 |
| --- | --- | --- |
| 2 | 1.000974 | 1.081148 |
| 3 | 1.000974 | 1.800725 |
| 4 | 1.085339 | 0.784761 |
| 5 | 1.000974 | 1.626929 |
| 6 | 1.116335 | 2.18263 |
| 7 | 1.000974 | 0.664538 |
| 8 | 1.085339 | 0.884114 |
| 9 | 1.000974 | 0.4959 |
| 10 | 1.116335 | 1.0886 |
| 11 | 1.000974 | 4.1174 |
| 12 | 1.085339 | 6.608539 |
| 13 | 1.000974 | 14.17255 |
| 14 | 1.116335 | 0.095867 |
| 15 | 1.416959 | 21.352 |
| 16 | 1.085339 | 0.1995 |
| 17 | 1.020974 | 0.331005 |
| 18 | 1.100974 | 1.80753 |
| 19 | 1.116335 | 39.58893 |
| 20 | 1.416959 | 7.965785 |
| 21 | 2.085339 | 0.31107 |
| 22 | 1.852432 | 0.503633 |
| 23 | 1.909226 | 0.534963 |
| 24 | 2.373662 | 17.67681 |
| 25 | 2.13138 | 0.331005 |
| 26 | 0.550974 | 1.80753 |
| 27 | 1.116335 | 39.58893 |
| 28 | 1.416959 | 7.965785 |
| 29 | 1.085339 | 0.31107 |
| 30 | 1.852432 | 0.503633 |
| 31 | 1.909226 | 0.534963 |
| 32 | 2.373662 | 17.67681 |

**Table S3-2 Relative gray value of CD73 protein expression in 32 pairs of GC tissues**

| 1 | 0.047707 | 1.214452 |
| --- | --- | --- |
| 2 | 0.601428 | 1.083241 |
| 3 | 0.693818 | 0.992933 |
| 4 | 0.846486 | 0.883245 |
| 5 | 0.847353 | 1.008153 |
| 6 | 0.882617 | 0.661507 |
| 7 | 0.884376 | 1.050115 |
| 8 | 0.911752 | 1.219856 |
| 9 | 1.017831 | 1.453886 |
| 10 | 1.064846 | 0.952105 |
| 11 | 1.088483 | 1.397182 |
| 12 | 1.143967 | 1.221672 |
| 13 | 0.74098 | 1.672231 |
| 14 | 2.022408 | 1.53511 |
| 15 | 1.487725 | 2.529893 |
| 16 | 4.632425 | 2.673786 |
| 17 | 0.571924 | 1.238659 |
| 18 | 0.757394 | 0.608466 |
| 19 | 1.362134 | 0.94088 |
| 20 | 1.189605 | 0.805563 |
| 21 | 1.853213 | 1.734444 |
| 22 | 0.868508 | 1.242671 |
| 23 | 1.257047 | 1.789223 |
| 24 | 2.007655 | 2.698422 |
| 25 | 1.043529 | 1.739938 |
| 26 | 1.280278 | 1.799714 |
| 27 | 1.572457 | 2.954189 |
| 28 | 1.514054 | 3.121142 |
| 29 | 1.107796 | 0.803734 |
| 30 | 0.532497 | 0.763434 |
| 31 | 0.720463 | 1.136846 |
| 32 | 1.125192 | 1.262315 |

**Table S3-3 S**urvival analysis of 116 GC patients in Nanfang cohort

| survival（days） | CD73-low(n=64) | CD73-high(n=107) |
| --- | --- | --- |
| 1110 | 0 |  |
| 1440 | 0 |  |
| 1050 | 0 |  |
| 1470 | 0 |  |
| 1260 | 0 |  |
| 1020 | 0 |  |
| 1230 | 0 |  |
| 1380 | 0 |  |
| 1350 | 0 |  |
| 1440 | 0 |  |
| 1410 | 0 |  |
| 1140 | 0 |  |
| 1290 | 0 |  |
| 1050 | 0 |  |
| 1200 | 0 |  |
| 1470 | 0 |  |
| 1170 | 0 |  |
| 1290 | 0 |  |
| 1080 | 0 |  |
| 1020 | 0 |  |
| 1470 | 0 |  |
| 1200 | 0 |  |
| 1440 | 0 |  |
| 1260 | 0 |  |
| 1170 | 0 |  |
| 1050 | 0 |  |
| 1050 | 0 |  |
| 1290 | 0 |  |
| 1290 | 0 |  |
| 1260 | 0 |  |
| 1230 | 0 |  |
| 1290 | 0 |  |
| 1380 | 0 |  |
| 1260 | 0 |  |
| 1320 | 0 |  |
| 1290 | 0 |  |
| 1470 | 0 |  |
| 1470 | 0 |  |
| 1470 | 0 |  |
| 1200 | 0 |  |
| 1440 | 0 |  |
| 1440 | 0 |  |
| 1170 | 0 |  |
| 1260 | 0 |  |
| 1260 | 0 |  |
| 1050 | 0 |  |
| 1470 | 0 |  |
| 1050 | 1 |  |
| 360 | 1 |  |
| 630 | 1 |  |
| 270 | 1 |  |
| 540 | 1 |  |
| 810 | 1 |  |
| 720 | 1 |  |
| 720 | 1 |  |
| 750 | 1 |  |
| 480 | 1 |  |
| 540 | 1 |  |
| 540 | 1 |  |
| 450 | 1 |  |
| 780 | 1 |  |
| 600 | 1 |  |
| 420 | 1 |  |
| 630 | 1 |  |
| 1440 |  | 0 |
| 1170 |  | 0 |
| 1080 |  | 0 |
| 990 |  | 0 |
| 1050 |  | 0 |
| 1170 |  | 0 |
| 1170 |  | 0 |
| 1170 |  | 0 |
| 1050 |  | 0 |
| 1440 |  | 0 |
| 1320 |  | 0 |
| 990 |  | 0 |
| 1380 |  | 0 |
| 1020 |  | 0 |
| 1320 |  | 0 |
| 840 |  | 0 |
| 1110 |  | 0 |
| 1200 |  | 0 |
| 1020 |  | 0 |
| 990 |  | 0 |
| 1440 |  | 0 |
| 1080 |  | 0 |
| 1050 |  | 0 |
| 1470 |  | 0 |
| 1440 |  | 0 |
| 1290 |  | 0 |
| 1050 |  | 0 |
| 1050 |  | 0 |
| 1290 |  | 0 |
| 1200 |  | 0 |
| 1020 |  | 0 |
| 1440 |  | 0 |
| 1170 |  | 0 |
| 1440 |  | 0 |
| 1470 |  | 0 |
| 1230 |  | 0 |
| 1320 |  | 0 |
| 1020 |  | 0 |
| 1200 |  | 0 |
| 1440 |  | 0 |
| 1170 |  | 0 |
| 1290 |  | 0 |
| 1080 |  | 0 |
| 1020 |  | 0 |
| 1080 |  | 0 |
| 1260 |  | 0 |
| 1020 |  | 0 |
| 1080 |  | 0 |
| 1080 |  | 0 |
| 1230 |  | 0 |
| 1020 |  | 0 |
| 900 |  | 0 |
| 1290 |  | 0 |
| 900 |  | 0 |
| 1050 |  | 0 |
| 1080 |  | 0 |
| 1170 |  | 0 |
| 840 |  | 0 |
| 840 |  | 0 |
| 1050 |  | 0 |
| 1080 |  | 1 |
| 750 |  | 1 |
| 540 |  | 1 |
| 120 |  | 1 |
| 1050 |  | 1 |
| 720 |  | 1 |
| 720 |  | 1 |
| 210 |  | 1 |
| 720 |  | 1 |
| 180 |  | 1 |
| 60 |  | 1 |
| 210 |  | 1 |
| 300 |  | 1 |
| 210 |  | 1 |
| 210 |  | 1 |
| 480 |  | 1 |
| 600 |  | 1 |
| 810 |  | 1 |
| 330 |  | 1 |
| 930 |  | 1 |
| 900 |  | 1 |
| 300 |  | 1 |
| 690 |  | 1 |
| 240 |  | 1 |
| 930 |  | 1 |
| 390 |  | 1 |
| 450 |  | 1 |
| 690 |  | 1 |
| 420 |  | 1 |
| 810 |  | 1 |
| 900 |  | 1 |
| 180 |  | 1 |
| 570 |  | 1 |
| 300 |  | 1 |
| 510 |  | 1 |
| 600 |  | 1 |
| 660 |  | 1 |
| 720 |  | 1 |
| 810 |  | 1 |
| 510 |  | 1 |
| 630 |  | 1 |
| 1050 |  | 1 |
| 300 |  | 1 |
| 540 |  | 1 |
| 480 |  | 1 |
| 450 |  | 1 |
| 1140 |  | 1 |

**Table S3-4 Relative mRNA expression of CD73 in GC cells**

| relative fold change | MKN45 |  |  | AGS |  |
| --- | --- | --- | --- | --- | --- |
|  | NC | siCD73-949 | siCD73-1070 | Vector | CD73 |
| replication1 | 28.8400531 | 5.31476034 | 1 | 1 | 12.7285837 |
| replication2 | 44.3235566 | 7.16020649 | 1.10956336 | 1.02811383 | 20.5348144 |
| replication3 | 24.4201707 | 4.78992905 | 5.13370249 | 1.70526978 | 15.6707248 |

**Table S3-5 T**ranswell analysis raw data

| **Figure2** | MKN45 |  | BGC823 |  | AGS |  | MGC803 |  |
| --- | --- | --- | --- | --- | --- | --- | --- | --- |
| migration cells number | NC | siCD73 | NC | siCD73 | Vector | CD73 | Vector | CD73 |
| field1 | 436 | 203 | 520 | 265 | 67 | 117 | 342 | 480 |
| field2 | 377 | 216 | 572 | 252 | 73 | 125 | 324 | 580 |
| field3 | 410 | 227 | 652 | 248 | 72 | 109 | 452 | 416 |
| field4 | 385 | 219 | 432 | 268 | 69 | 113 | 408 | 660 |
| field5 | 405 | 253 | 444 | 312 | 86 | 142 | 380 | 652 |
|  |  |  |  |  |  |  |  |  |
| **Figure3** | MKN45 |  |  |  |  | AGS |  |  |
| migration cells | LV-NC/Vector | CD73-RNAi/Vector | CD73-RNAi/c-Jun |  | migration cells | LV-NC | LV-CD73 | LV-CD73/SP600125 |
| field1 | 110 | 67 | 99 |  | field1 | 97 | 142 | 46 |
| field2 | 138 | 60 | 77 |  | field2 | 60 | 131 | 51 |
| field3 | 111 | 54 | 94 |  | field3 | 88 | 127 | 47 |
| field4 | 133 | 50 | 74 |  | field4 | 72 | 153 | 56 |
| field5 | 117 | 65 | 94 |  | field5 | 86 | 138 | 46 |
|  |  |  |  |  |  |  |  |  |
| **Figure5** | AGS |  |  |  |  |  |  |  |
| migration cells | LV-NC | LV-CD73 | LV-CD73/siRICS |  |  |  |  |  |
| field1 | 97 | 142 | 46 |  |  |  |  |  |
| field2 | 60 | 131 | 51 |  |  |  |  |  |
| field3 | 88 | 127 | 47 |  |  |  |  |  |
| field4 | 72 | 153 | 56 |  |  |  |  |  |
| field5 | 86 | 138 | 46 |  |  |  |  |  |
|  |  |  |  |  |  |  |  |  |

Table S3-6 **Wound healing assays analysis raw data**

| **Figure2** | MKN45 |  | BGC823 |  | AGS |  | MGC803 |  |
| --- | --- | --- | --- | --- | --- | --- | --- | --- |
| relative migration area | NC | siCD73 | NC | siCD73 | Vector | CD73 | Vector | CD73 |
| field 1 | 0.8971962 | 0.5207668 | 0.8796992 | 0.6162362 | 0.3622642 | 0.8639456 | 0.3790614 | 0.8526786 |
| field 2 | 0.8223684 | 0.4888889 | 0.8683274 | 0.6392857 | 0.4411765 | 0.9072165 | 0.3452381 | 0.7962963 |
| field 3 | 0.9009288 | 0.6091205 | 0.8992537 | 0.547619 | 0.3736655 | 0.8368794 | 0.2125984 | 0.8711111 |
| field 4 | 0.9413681 | 0.5665635 | 0.9260563 | 0.6305085 | 0.4134276 | 0.8986486 | 0.3012552 | 0.8644068 |
| field 5 | 0.9079365 | 0.550152 | 0.7847534 | 0.6313869 | 0.5591398 | 0.9285714 | 0.2439024 | 0.8538813 |
|  |  |  |  |  |  |  |  |  |
| **Figure3** | MKN45 |  |  |  |  | AGS |  |  |
| migration cells | LV-NC/Vector | CD73-RNAi/Vector | CD73-RNAi/c-Jun |  | migration cells | LV-NC | LV-CD73 | LV-CD73/SP600125 |
| field1 | 0.5705522 | 0.140884 | 0.3116147 |  | field1 | 0.3015873 | 0.4444444 | 0.2214765 |
| field2 | 0.5823529 | 0.1994535 | 0.3522727 |  | field2 | 0.1854305 | 0.5034965 | 0.3151125 |
| field3 | 0.4817073 | 0.1830239 | 0.2893983 |  | field3 | 0.2556634 | 0.4551724 | 0.2191781 |
| field4 | 0.4845679 | 0.121813 | 0.32493 |  | field4 | 0.176282 | 0.4265734 | 0.1768953 |
| field5 | 0.374613 | 0.09014084 | 0.2985507 |  | field5 | 0.3645161 | 0.4042553 | 0.1790541 |
|  |  |  |  |  |  |  |  |  |
| **Figure5** | AGS |  |  |  |  |  |  |  |
| relative migration area | LV-NC | LV-CD73 | LV-CD73/siRICS |  |  |  |  |  |
| field1 | 0.3015873 | 0.4444444 | 0.2214765 |  |  |  |  |  |
| field2 | 0.1854305 | 0.5034965 | 0.3151125 |  |  |  |  |  |
| field3 | 0.2556634 | 0.4551724 | 0.2191781 |  |  |  |  |  |
| field4 | 0.176282 | 0.4265734 | 0.1768953 |  |  |  |  |  |
| field5 | 0.3645161 | 0.4042553 | 0.1790541 |  |  |  |  |  |

Table S3-7 **Relative gray value analysis raw data**

| **Figure3** | MKN45 |  |  |  |  |  |  |  |  |
| --- | --- | --- | --- | --- | --- | --- | --- | --- | --- |
|  | LV-NC/Vector |  |  | CD73-RNAi/Vector |  |  | CD73-RNAi/c-Jun |  |  |
| c-Jun | 0.614551 | 0.597889 | 0.60529 | 0.467559 | 0.473635 | 0.479766 | 1.097394 | 1.102116 | 1.086942 |
| CD73 | 1.878035 | 1.839548 | 1.834926 | 1.660227 | 1.642212 | 1.631141 | 3.504373 | 3.563969 | 3.467069 |
| E-cadherin | 1.840733 | 1.75497 | 1.792788 | 2.64203 | 2.57469 | 2.741057 | 2.240579 | 2.162476 | 2.20816 |
| N-cadherin | 1.674196 | 1.657918 | 1.632699 | 1.626753 | 1.645495 | 1.698714 | 2.413416 | 2.412308 | 2.373641 |
| Vimentin | 1.386682 | 1.375522 | 1.34771 | 0.972957 | 0.971785 | 1.023472 | 1.194532 | 1.196602 | 1.178929 |
| β-catenin | 1.839484 | 1.811269 | 1.796272 | 2.083768 | 2.053972 | 2.154926 | 2.961388 | 3.054092 | 2.934965 |
|  |  |  |  |  |  |  |  |  |  |
| **Figure3** | AGS |  |  |  |  |  |  |  |  |
|  | LV-NC |  |  | LV-CD73 |  |  | LV-CD73/SP600125 |  |  |
| c-Jun | 0.686823 | 0.672524 | 0.703119 | 1.385091 | 1.395899 | 1.34594 | 1.683791 | 1.649259 | 1.693453 |
| CD73 | 1.514233 | 1.489198 | 1.548078 | 2.012707 | 2.05241 | 1.965006 | 1.361318 | 1.344158 | 1.355475 |
| E-cadherin | 1.778704 | 1.704161 | 1.904384 | 1.840856 | 1.81552 | 1.747206 | 3.050949 | 2.945733 | 3.036116 |
| N-cadherin | 1.522408 | 1.512216 | 1.592911 | 2.190393 | 2.201998 | 2.129596 | 1.846044 | 1.854249 | 1.814446 |
| Vimentin | 1.614117 | 1.576685 | 1.758912 | 2.386104 | 2.42696 | 2.271606 | 2.616471 | 2.619176 | 2.568683 |
| β-catenin | 1.40571 | 1.39288 | 1.439926 | 1.91166 | 1.949961 | 1.859438 | 1.867608 | 1.847323 | 1.873996 |
|  |  |  |  |  |  |  |  |  |  |
| **Figure4** | LV-NC | LV-CD73 | LV-NC | CD73-RNAi | |  |  |  |  |
| CD73 | 0.71661 | 1.37359 | 1.586146 | 1.055158 |  |  |  |  |  |
| RICS | 1.214579 | 1.623915 | 1.094189 | 0.95198 |  |  |  |  |  |
| β-catenin | 0.393727 | 0.987138 | 1.532905 | 1.015918 |  |  |  |  |  |
| E-cadherin | 1.383252 | 1.00886 | 0.938982 | 2.237425 |  |  |  |  |  |
| Vimentin | 0.370604 | 0.803896 | 0.985241 | 0.560719 |  |  |  |  |  |
| Snail | 1.060271 | 1.911778 | 1.804569 | 0.744873 |  |  |  |  |  |
| p-LIMK | 1.437448 | 1.197045 | 1.025678 | 1.841057 |  |  |  |  |  |
| p-cofilin | 1.325398 | 0.91714 | 0.478361 | 1.816009 |  |  |  |  |  |
|  |  |  |  |  |  |  |  |  |  |
| **Figure6** | MKN45 |  |  |  |  |  |  |  |  |
| relative gray value | NC | | | siCD73 | | | NC/APCP | | |
| CD73 | 2.497831 | 2.505086 | 2.512123 | 1.509914 | 1.56269 | 1.574852 | 1.140045 | 1.13676 | 1.164166 |
| RICS | 2.462792 | 2.613957 | 2.540824 | 2.041401 | 1.963203 | 2.059132 | 2.19844 | 2.109946 | 2.188332 |
| p-LIMK | 1.925524 | 1.844618 | 1.995369 | 2.581482 | 2.720619 | 2.616942 | 2.527507 | 2.520242 | 2.547175 |
| p-cofilin | 1.272201 | 1.401323 | 1.425913 | 2.458589 | 2.287448 | 2.319076 | 2.33276 | 2.21783 | 2.322406 |
|  |  |  |  |  |  |  |  |  |  |
| **Figure6** | AGS |  |  |  |  |  |  |  |  |
| relative gray value | Vector | | | CD73 | | | CD73+APCP | | |
| CD73 | 0.837431 | 0.801947 | 0.797089 | 1.087474 | 1.190509 | 1.039157 | 1.094812 | 1.071318 | 1.032627 |
| RICS | 0.748842 | 0.703332 | 0.754283 | 1.014726 | 1.31734 | 1.005434 | 0.842053 | 0.709504 | 0.876731 |
| p-LIMK | 1.283792 | 1.216586 | 1.289975 | 1.002791 | 0.901377 | 0.989466 | 1.289578 | 1.091571 | 1.336847 |
| p-cofilin | 1.092688 | 1.026939 | 1.072772 | 0.489825 | 0.605062 | 0.555342 | 1.031992 | 0.890459 | 0.992407 |

Table S3-8 **Analysis raw data in Figure7**

| **Figure7** |  |  |  |  |  |  |  |  |  |  |
| --- | --- | --- | --- | --- | --- | --- | --- | --- | --- | --- |
| number of metastasis nodules | LV-NC | CD73-RNAi |  |  | relative metastasis area | LV-NC | CD73-RNAi |  |  |  |
| mouse1 | 93 | 69 |  |  | mouse1 | 1489.765 | 1552.785 |  |  |  |
| mouse2 | 79 | 79 |  |  | mouse2 | 1457.586 | 631.0534 |  |  |  |
| mouse3 | 99 | 16 |  |  | mouse3 | 1423.457 | 132.3187 |  |  |  |
| mouse4 | 73 | 12 |  |  | mouse4 | 1318.441 | 332.6779 |  |  |  |
| mouse5 | 93 | 10 |  |  | mouse5 | 1424.454 | 93.98744 |  |  |  |
|  |  |  |  |  |  |  |  |  |  |  |
|  | Peritoneal | | gastric | | intestinal | | hepatic | | spleenic | |
| number of metastasis nodules | LV-NC | CD73-RNAi | LV-NC | CD73-RNAi | LV-NC | CD73-RNAi | LV-NC | CD73-RNAi | LV-NC | CD73-RNAi |
| mouse1 | 7 | 1 | 6 | 2 | 68 | 57 | 12 | 7 | 0 | 2 |
| mouse2 | 3 | 1 | 10 | 3 | 52 | 63 | 5 | 10 | 9 | 2 |
| mouse3 | 11 | 1 | 9 | 5 | 68 | 8 | 5 | 0 | 6 | 2 |
| mouse4 | 11 | 1 | 6 | 2 | 49 | 4 | 4 | 0 | 3 | 5 |
| mouse5 | 6 | 3 | 9 | 1 | 70 | 5 | 8 | 0 | 0 | 1 |
