# Supplementary material for: CD73 promotes tumor metastasis by modulating RICS/RhoA signaling and EMT in gastric cancer
Source: Cell Death Dis. 2020 Mar 23;11(3):202. doi: 10.1038/s41419-020-2403-6 (PMC7089986; doi:10.1038/s41419-020-2403-6)
Supplement: Supplementary file 9 — Reproducibility Checklist [file 41419_2020_2403_MOESM9_ESM.pdf]

Corresponding Author Name: Hao Liu
Manuscript Number: CDDIS-19-3882R

nature publishing group npg

# Reporting Checklist

This checklist is used to ensure good reporting standards and to improve the reproducibility of published results. **Please respond completely to all questions relevant to your manuscript.** For more information, please read the journal's Guide to Authors.

■ Check here to confirm that the following information is available in the Material & Methods section:

- the **exact sample size (*n*)** for each experimental group/condition, given as a number, not a range;
- a **description of the sample collection** allowing the reader to understand whether the samples represent **technical or biological replicates** (including how many animals, litters, culture, etc.);
- a **statement of how many times the experiment shown was replicated in the laboratory**;
- **definitions of statistical methods and measures**: (For small sample sizes (n<5) descriptive statistics are not appropriate, instead plot individual data points)
  - very common tests, such as *t*-test, simple $\chi^2$ tests, Wilcoxon and Mann-Whitney tests, can be unambiguously identified by name only, but more complex techniques should be described in the methods section;
  - are tests one-sided or two-sided?
  - are there adjustments for multiple comparisons?
  - **statistical test results**, e.g., ***P* values**;
  - definition of **'center values'** as **median or mean**;
  - definition of **error bars as s.d. or s.e.m. or c.i.**

Please ensure that the answers to the following questions are reported **in the manuscript itself.** We encourage you to include a specific subsection in the methods section for statistics, reagents and animal models. Below, provide the page number or section and paragraph number.

| **Statistics and general methods** | **Reported in section/paragraph or page #** |
|---|---|
| 1. How was the sample size chosen to ensure adequate power to detect a pre-specified effect size? (Give section/paragraph or page #) | Page20-21 Materials and Methods /Statistical analysis Sample size was chosen according to previous observations, which perform similar experiments to see significant results, or the results from our preliminary [illegible] |
| For animal studies, include a statement about sample size estimate even if no statistical methods were used. | Page20-21 Materials and Methods /Statistical analysis For animal studies, sample size was estimated to be no less than 5 in seeding or hematogenous metastasis models |
| 2. Describe inclusion/exclusion criteria if samples or animals were excluded from the analysis. Were the criteria pre-established? (Give section/paragraph or page #) | Not Applicable |
| 3. If a method of randomization was used to determine how samples/animals were allocated to experimental groups and processed, describe it. (Give section/paragraph or page #) | Page 18Materials and Methods / In vivo metastasis assay Animals were allocated to experimental and control group according to a random number table and no blinding was done. |
| For animal studies, include a statement about randomization even if no randomization was used. | Page 18 Materials and Methods /In vivo metastasis assay Animals were allocated to experimental and control group according to a random number table and no blinding was [illegible] |

| | |
|---|---|
| 4. If the investigator was blinded to the group allocation during the experiment and/or when assessing the outcome, state the extent of blinding. (Give section/paragraph or page #) | Page 18 Materials and Methods /In vivo metastasis assay<br>Animals were allocated to experimental and control group according to a random number table and no blinding was done. |
| For animal studies, include a statement about blinding even if no blinding was done. | Page 18 Materials and Methods /In vivo metastasis assay<br>Animals were allocated to experimental and control group according to a random number table and no blinding was |
| 5. For every figure, are statistical tests justified as appropriate? | Page20-21 Materials and Methods /Statistical analysis<br>The Student t test was used to detect significance between groups and 2 test was used for measurement data. |
| Do the data meet the assumptions of the tests (e.g., normal distribution)? | Page20-21 Materials and Methods /Statistical analysis<br>The Student t test was used to detect significance between groups and 2 test was used for measurement data.<br>[illegible] |
| Is there an estimate of variation within each group of data? | Page20-21Materials and Methods / Statistical analysis<br>ANOVA analysis was used for analysis of differences among three or more groups and post-hoc analysis was performed. |
| Is the variance similar between the groups that are being statistically compared? (Give section/paragraph or page #) | Page20-21 Materials and Methods /Statistical analysis<br>ANOVA analysis was used for analysis of differences among three or more groups and post-hoc analysis was performed. Multiple comparison was done after<br>[illegible] |

| **Reagents** | **Reported in section/paragraph or page #** |
|---|---|
| 6. Report the source of antibodies (vendor and catalog number) | Page17Materials and Methods / Immunofluorescence(IF) assays<br>antibodies (CD73, abcam, ab81720 1:50, E-cadherin, |
| 7. Identify the source of cell lines and report if they were recently authenticated (e.g., by STR profiling) and tested for mycoplasma contamination | Page 16Materials and Methods /cell lines and cell culture<br>Human gastric cancer cell lines MKN45, SGC7901, AGS, MGC803, BGC823 and human gastric mucosal cell line GES-1 were obtained from American Type Culture Collection (ATCC, Manassas, VA, USA), generated, in the Department of General Surgery, Nanfang Hospital, and |

| **Animal Models** | **Reported in section/paragraph or page #** |
|---|---|
| 8. Report species, strain, sex and age of animals | Page 18Materials and Methods / In vivo metastasis assay<br>Five-week-old male BALB/c nude mice were purchased |
| 9. For experiments involving live vertebrates, include a statement of compliance with ethical regulations and identify the committee(s) approving the experiments. | Not Applicable |

10. We recommend consulting the ARRIVE guidelines (*PLoS Biol.* **8**(6), e1000412,2010) to ensure that other relevant aspects of animal studies are adequately reported.

## Human subjects

**Reported in section/paragraph or page #**

| Item | Reported in section/paragraph or page # |
| --- | --- |
| 11. Identify the committee(s) approving the study protocol. | Page 15-16 Materials and Methods /Patients and specimens Collection of human tissues and analysis of clinical data was approved by the Ethics Committee of Nanfang Hospital. |
| 12. Include a statement confirming that informed consent was obtained from all subjects. | Page 15-16 Materials and Methods /Patients and specimens Collection of human tissues and analysis of clinical data was approved by the Ethics Committee of Nanfang Hospital. All |
| 13. For publication of patient photos, include a statement confirming that consent to publish was obtained. | Not Applicable |
| 14. Report the clinical trial registration number (at ClinicalTrials.gov or equivalent). | Not Applicable |

15. For phase II and III randomized controlled trials, please refer to the CONSORT statement and submit the CONSORT checklist with your submission.

16. For tumor marker prognostic studies, we recommend that you follow the REMARK reporting guidelines.

## Data deposition

| Item | Reported in section/paragraph or page # |
| --- | --- |
| 17. Provide accession codes for deposited data. Data deposition in a public repository is mandatory for:<br>a. Protein, DNA and RNA sequences<br>b. Macromolecular structures<br>c. Crystallographic data for small molecules<br>d. Microarray data | Page 21 Supplementary information<br>Expression profiling data of TCGA in this study was downloaded from http://cancergenome.nih.gov/ |

Deposition is strongly recommended for many other datasets for which structured public repositories exist; more details on our data policy are available in the Guide to Authors. We encourage the provision of other source data in supplementary information or in unstructured repositories such as Figshare and Dryad. We encourage publication of Data Descriptors (see Scientific Data) to maximize data reuse.

| Item | Reported in section/paragraph or page # |
| --- | --- |
| 18. If computer code was used to generate results that are central to the paper's conclusions, include a statement in the Methods section under "**Code availability"** to indicate whether and how the code can be accessed. Include version information as necessary and any restrictions on availability. | Not Applicable |
