# Supplementary material for: CD73 promotes tumor metastasis by modulating RICS/RhoA signaling and EMT in gastric cancer
Source: Cell Death Dis. 2020 Mar 23;11(3):202. doi: 10.1038/s41419-020-2403-6 (PMC7089986; doi:10.1038/s41419-020-2403-6)
Supplement: Supplementary file 10 — Supplementary file cddis-author-contribution-form.pdf [file 41419_2020_2403_MOESM10_ESM.pdf]

DECLARATION OF CONTRIBUTIONS TO ARTICLE

ADMC

Manuscript Number:	Journal Name:	
CD73 promotes tumor metastasis by modulating RICS/RhoA signaling and EMT in gastric cancer	*Cell Death & Disease*	(the 'Journal')

Proposed Title of the Contribution:

design experiment，perform experiment, write manuscript, statistical analysis, generate data, label image, polish language , create charts, bioinformatic analysis (the 'Contribution)

Author(s):

Zhijun Xu, Chuncai Gu, Xingxing Yao, Weihong Guo, Huanan Wang, Tian Lin, Fengping Li, Da Chen, Jianhua Wu, Gengtai Ye, Liying Zhao, Yanfeng Hu, Jiang Yu, Jiaolong Shi, Guoxin Li, Hao Liu (the 'Authors')

For all *CDDis* articles, each person named as an author in the published version must be able to show he or she has contributed substantially to the article.

Authorship credit should be based on 1) substantial contributions to conception and design, acquisition of data, or analysis and interpretation of data; 2) drafting the article or revising it critically for important intellectual content; and 3) final approval of the version to be published. Authors should meet conditions 1, 2 and 3.

Any person who cannot be shown to have made a substantial contribution to the article cannot be listed as an author in the final version. The name of any person who is deemed to have made a minor contribution can, however, appear in the Acknowledgments section of the article.

Please complete the table below to indicate the contributions of all named authors to the manuscript.

Author Full Name:	Specification of Contribution to the Manuscript:
Xu Zhijun	XZ designed，performed the experiment and wrote manuscript.
Gu Chuncai	GC helped write manuscript and performed bioinformatic analysis.
Xingxing Yao	YX performed statistical analysis and prepared the graph.
Weihong Guo	GW generated clinical samples and data.
Huanan Wang	WH gave help in cell culture and treatment.
Tian Lin	LT performed animal experiments.
Fengping Li	LFP performed molecular experiments.
Da Chen,Jianhua Wu	CD and WJ performed IHC and IF experiments.
Gengtai Ye,Liying Zhao, Yanfeng Hu	YG, ZL and HY collected tissue samples and clinical data for TMA.
Jiang Yu	YJ monitored and provided help in access of clinical database.
Jiaolong Shi	SJ wrote the manuscript and polished language of the manuscript.
Guoxin Li	LG monitored the experiments and provided help in design.
Hao Liu	LH designed the experiments.

Please complete the table below to indicate the contributions of all named authors to the figures.

Figure 1:

In Figure1, XZ generated the data and prepared panel A, B. GW generated the data and prepared panel C,D. WJ and CD helped generated information of panel C,D.

Figure 2:

In Figure2, GC generated data and prepared panel A, B, C and YX prepared panel D.

Figure 3:

In Figure3, YX generated data and prepared panel A,C. GW prepared panel E,F.

Figure 4:

In Figure 4, XZ prepared data in panel A,B,D,E and GC generated data in panel C .

Figure 5:

In Figure 5, XZ generated data and prepared panel A, B,C.

Figure 6:

In Figure 6, LF generated data and prepared panel A,B,C.
In Figure7, LT prepared tumor xenograft models. YX prepared the panel E.

Signed for and on behalf of the Author(s):	Print Name:	Date:
Liu Hao	Liu Hao	2019.10.29
